# What is the association of hypothyroidism with risks of cardiovascular events and mortality? A meta-analysis of 55 cohort studies involving 1,898,314 participants

**DOI:** 10.1186/s12916-017-0777-9

**Published:** 2017-02-02

**Authors:** Yu Ning, Yun J. Cheng, Li J. Liu, Jaskanwal D. S. Sara, Zhi Y. Cao, Wei P. Zheng, Tian S. Zhang, Hui J. Han, Zhen Y. Yang, Yi Zhang, Fei L. Wang, Rui Y. Pan, Jie L. Huang, Ling L. Wu, Ming Zhang, Yong X. Wei

**Affiliations:** 1 Department of Cardiology, Beijing Anzhen Hospital, Capital Medical University, Beijing, China; 2 Department of Cardiology, the Eastern Hospital of the First Affiliated Hospital, Sun Yat-Sen University, Guangzhou, China; 3 Division of Cardiovascular Diseases, Mayo Clinic, Rochester, MN USA; 4 Department of Cardiology, Changzheng Hospital, Second Military Medical University, Shanghai, China; 5 Department of Cardiology, Provincial Clinical Medical College of Fujian Medical University, Fuzhou, China; 6 Department of TCM, Jing’An District Centre Hospital, Shanghai, China; 7 Department of Epidemiology and Biostatistics, Institute of Basic Medical Sciences Chinese Academy of Medical Sciences, School of Basic Medicine Peking Union Medical College, Beijing, China; 8 Key Laboratory of Trace Element Nutrition, Ministry of Health of China; National Institute for Nutrition and Health, Chinese Center for Disease Control and Prevention, Beijing, China; 9 Department of Endocrinology, Quanzhou First Hospital, Fujian Medical University, Quanzhou, China; 10 Department of Pharmacology, Beijing Anzhen Hospital, Beijing Institute of Heart Lung and Blood Vessel Diseases, Capital Medical University, Beijing, China; 11 Department of Cardiology, Guangdong General Hospital, Southern Medical University, Guangzhou, China; 12 Department of Respiration, Zhujiang Hospital of Southern Medical University, Guangzhou, China; 13 Department of Otolaryngology Head & Neck Surgery, Beijing Anzhen Hospital, Capital Medical University, No.2, Anzhen Road, Chaoyang District, 100029 Beijing, China

**Keywords:** Hypothyroidism, Cardiovascular events, Mortality, Meta-analysis

## Abstract

**Background:**

Whether hypothyroidism is an independent risk factor for cardiovascular events is still disputed. We aimed to assess the association between hypothyroidism and risks of cardiovascular events and mortality.

**Methods:**

We searched PubMed and Embase from inception to 29 February 2016. Cohort studies were included with no restriction of hypothyroid states. Priori main outcomes were ischemic heart disease (IHD), cardiac mortality, cardiovascular mortality, and all-cause mortality.

**Results:**

Fifty-five cohort studies involving 1,898,314 participants were identified. Patients with hypothyroidism, compared with euthyroidism, experienced higher risks of IHD (relative risk (RR): 1.13; 95% confidence interval (CI): 1.01–1.26), myocardial infarction (MI) (RR: 1.15; 95% CI: 1.05–1.25), cardiac mortality (RR: 1.96; 95% CI: 1.38–2.80), and all-cause mortality (RR: 1.25; 95% CI: 1.13–1.39); subclinical hypothyroidism (SCH; especially with thyrotropin level ≥10 mIU/L) was also associated with higher risks of IHD and cardiac mortality. Moreover, cardiac patients with hypothyroidism, compared with those with euthyroidism, experienced higher risks of cardiac mortality (RR: 2.22; 95% CI: 1.28–3.83) and all-cause mortality (RR: 1.51; 95% CI: 1.26–1.81).

**Conclusions:**

Hypothyroidism is a risk factor for IHD and cardiac mortality. Hypothyroidism is associated with higher risks of cardiac mortality and all-cause mortality compared with euthyroidism in the general public or in patients with cardiac disease.

**Electronic supplementary material:**

The online version of this article (doi:10.1186/s12916-017-0777-9) contains supplementary material, which is available to authorized users.

## Background

Thyroid hormones have wide effects on the body and play an important role in homeostasis of the cardiovascular system [1]. Abnormal thyroid function has health consequences for the general public. Patients with hypothyroidism have an increased risk of cardiovascular abnormalities, such as accelerated atherosclerosis [2].

Whether hypothyroidism (“hypothyroidism” refers to the combination of subclinical hypothyroidism (SCH) and overt hypothyroidism (OHypo) in this article), SCH or OHypo, is an independent risk factor for cardiovascular events or mortality has conflicting opinions. It is also conflicting whether adults with hypothyroidism and preexisting cardiovascular disease might be at particularly high cardiovascular risk [3–5]. Although there have been meta-analyses and even individual participant data (IPD) meta-analyses on the subject so far [6–10], they have been restricted to patients with SCH. Moreover, many additional cohort studies have been published since. Therefore, a comprehensive updated meta-analysis is warranted.

## Methods

### Search strategy

This meta-analysis follows the Preferred Reporting Items for Systematic Reviews and Meta-Analyses (PRISMA) Checklist (Additional file 1). We searched PubMed and Embase from inception to 29 February 2016, using the following text and key words, both as MeSH terms and text words: hypothyroidism, subclinical hypothyroidism, thyroid diseases, thyroid function, thyroid status, coronary heart diseases, ischemic heart diseases, myocardial infarction, coronary atherosclerosis, cardiovascular diseases, death, mortality, heart failure, stroke, atrial fibrillation, arrhythmia, peripheral artery diseases. We searched articles published in any language and screened references from these studies to identify other relevant studies. YN and YJC performed the literature search independently. Differences were resolved by discussion.

### Inclusion criteria

To minimize differences, studies were required (1) to measure thyroid function and follow persons prospectively; to assess ischemic heart disease (IHD), mortality or other cardiovascular events; and to provide risk estimates or sufficient data to calculate risk estimates associated with hypothyroidism compared with euthyroidism; (2) to be cohort studies published as original articles; and (3) to be independent. For multiple publications of the same study design, we included the estimates from the most recent or informative reports.

### Data collection and analysis

Data were extracted by one reviewer (YN) and independently checked by another three reviewers (YJC, LJL, and HJH); the data included the first author’s name, publication year, location, female proportion, mean age, mean follow-up years, sample size, study design, study population, number of cardiovascular events and mortality, risk estimates, categories and definitions of hypothyroidism, thyroid treatment, and covariates adjusted for in the multivariable analyses. Primary authors were contacted by email if the study did not provide the data needed. We used the Newcastle-Ottawa scale to evaluate the quality of cohort studies. In the present analyses, we regarded a study awarded a total score of ≥6 as a high-quality study [11].

Hypothyroidism was categorized as SCH and OHypo. Their definitions varied across studies. Generally, SCH was defined as elevated thyroid-stimulating hormone (TSH) levels and normal free tetraiodothyronine (FT4) levels. OHypo was defined as elevated TSH but decreased FT4 levels [5]. In this analysis, hypothyroidism refers to the combination of SCH and OHypo. The primary exposure of interest is hypothyroidism (i.e., participants with an increased TSH level regardless of FT4 level).

The primary outcomes were IHD, cardiac mortality, cardiovascular mortality, and all-cause mortality as defined by International Classification of Diseases-10-CM (ICD-10-CM) diagnosis codes. For IHD (I20–I25), we included angina pectoris, myocardial infarction (MI), and chronic ischemic heart disease. For cardiac mortality, we included deaths caused by IHD, progressive heart failure (HF), arrhythmia, or cardiac arrest (I20–I25; I44–I50). For cardiovascular mortality, we included deaths from diseases of the circulatory system (I00–I99). We used both cardiac and cardiovascular mortality for the following two reasons: first, both of them had been discussed in different meta-analyses previously; second, the related data of both outcomes were available from considerable original cohort studies. Therefore, it is necessary to analyze both of them in one meta-analysis to achieve comprehensive comparison to the previous meta-analyses. The secondary outcomes were stroke, HF, MI, atrial fibrillation (AF), and total cardiovascular events.

### Statistical analyses

The available risk estimates that were extracted were mostly hazard ratios (HRs), while those in partial studies were rate ratios (RRs) [12–15], incidence rate ratios (IRRs) [16, 17], or odds ratios (ORs) [18]. We used the most comprehensively adjusted estimates in the reports. When risk estimates and confidence intervals (CIs) were not provided [19–21], we calculated RRs and CIs from available data by using the Woolf method in Stata version 12.1 software (used for all analyses) [9, 22]. Since the absolute risk of IHD, cardiac mortality, cardiovascular mortality, all-cause mortality, stroke, HF, MI, AF, or total cardiovascular events is low, the four measures of association are expected to yield similar estimates of RR [23, 24]. Consequently, we estimated the pooled RRs and their CIs by using metan commands and random effects models based on the variance model developed by DerSimonian and Laird [9, 25]. We calculated the *I* ^2^ statistic to assess heterogeneity among studies. The formula is {(Q-df)/Q × 100%}, where Q is the chi-squared statistic and df is its degrees of freedom [26]. We use the following criteria: *I* ^2^ < 50%, low heterogeneity; 50–75%, moderate heterogeneity; >75%, high heterogeneity [26, 27].

Several predefined subgroup analyses were intended to explore potential sources of heterogeneity in primary outcomes (metan command); where enough studies (*n* ≥ 10) [28] were available, meta-regressions were conducted (metareg command). Specifically, we intended to conduct subgroup analyses by hypothyroid states and by study population to clarify whether adults with hypothyroidism and preexisting cardiovascular disease were at particularly high cardiovascular risk. Given previous findings of the possible protective effect of SCH in oldest old people [29], we intended to conduct subgroup analyses by mean age of populations. Predefined sensitivity analyses were performed to test the robustness of the results (metan command). When *I* ^2^ ≥ 50% (moderate or high heterogeneity), we used the hetred command in Stata to evaluate the change in between-study heterogeneity as one or more outlier studies were excluded from the calculations, a method which was developed by Patsopoulos [30]. If available, we intended to perform additional subgroup analyses of main outcomes by TSH levels (TSH ≥ 10.0 and TSH < 10.0 mIU/L) in SCH.

If there were at least 10 studies included in each outcome of the meta-analysis, publication bias or small study bias was assessed with a funnel plot by using Egger’s regression asymmetry test (metabias6 command) [31, 32]. Statistical tests were two-sided and used a significance level of *P* < 0.05.

## Results

### Search results

**Fig. 1:**
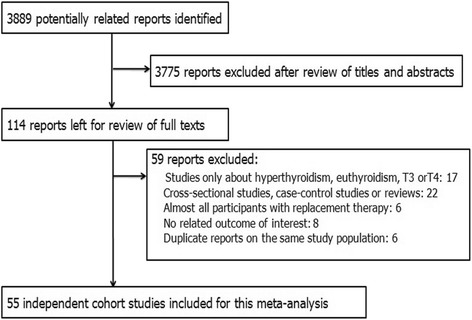
Flowchart of study selection

Of 3889 reports identified, we excluded 3775 studies that were unrelated to our theme and 59 studies after detailed evaluation. Fifty-five cohort studies met the inclusion criteria. Figure 1 shows details of the study selection.

Additional file 2: Table S1 shows the main characteristics of the 55 included studies. Thirty-two studies were population-based studies. Twenty-three studies were convenience samples (i.e., particular patient groups), of which 14 studies comprised cardiac patients. Nineteen studies analyzed with all hypothyroidism, 34 with SCH, and 8 with OHypo. Cohort studies included in each outcome are mainly prospective and mostly from Europe or North America. Thirty-seven of the 55 studies were published in the last 5 years (2012–2016). Of the 55 cohort studies, 49 studies were awarded a total score of ≥6 (Additional file 2: Table S2).

Additional file 2: Table S3 shows detailed information on adjustments in risk estimates of each cohort study. Most risk estimates were adjusted for age (47 studies) and sex (40 studies), which are well known as potential confounders of both thyroid disease status and risk of cardiovascular events. Forty studies (72.7%) reported an adjusted estimate for at least one of the main cardiovascular risk factors (body mass index (BMI), smoking, cholesterol, hypertension, and diabetes). Seven studies’ risk estimates were adjusted for replacement therapy (RT).

### Hypothyroidism and IHD

Thirteen studies with 615,596 individuals and 16,862 events analyzed with IHD. Twelve studies’ risk estimates were adjusted for age, ten adjusted for sex, and more than half adjusted for smoking, BMI, hypertension, or diabetes, but only three adjusted for RT.

**Fig. 2:**
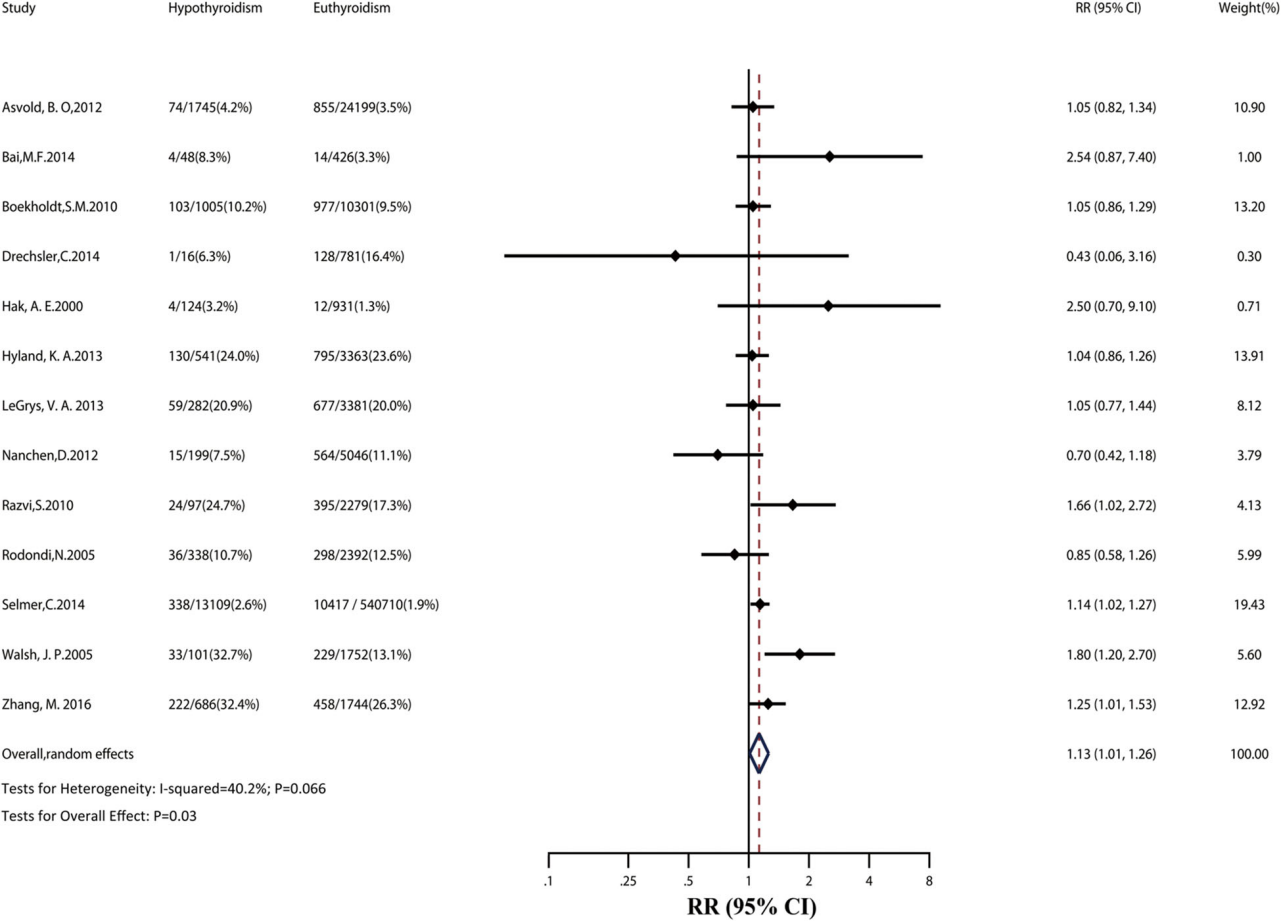
Relative risk (*RR*) of ischemic heart disease (*IHD*) associated with hypothyroidism compared with euthyroidism. The *dotted line* in forest plot represents pooled RR value in random effects model. *CI* confidence interval

Overall, patients with hypothyroidism experienced a higher risk of IHD compared to euthyroidism (RR: 1.13; 95% CI: 1.01–1.26), with evidence of low heterogeneity (*I* ^2^: 40.2%; *P* = 0.066; Fig. 2). Table 1 shows the results of sensitivity analyses. Risk estimates changed little after analyzing with fixed effects models, or after inclusion of studies with ≥10,000 participants, studies without RT at baseline, or high-quality studies. No publication bias was found (Egger test: *P* = 0.65; Additional file 2: Figure S1).

**Table 1:** Sensitivity analyses of pooled RRs of cardiovascular events and mortality

	IHD				Cardiac mortality				Cardiovascular mortality			
	*N* studies	RR (95% CI)	*I* ^2^	*P* value^a^	*N* studies	RR (95% CI)	*I* ^2^	*P* value^a^	*N* studies	RR (95% CI)	*I* ^2^	*P* value^a^
Statistical model												
Random effects	13	1.13 (1.01–1.26)	40.2%	0.07	7	1.96 (1.38–2.80)	66.2%	0.007	17	1.11 (0.97–1.28)	11.1%	0.32
Fixed effects	13	1.12 (1.05–1.20)	-	-	7	1.73 (1.45–2.08)	-	-	17	1.11 (0.99–1.25)	-	-
Analyses with												
Large cohort^b^	3	1.11 (1.01–1.21)	0.0%	0.70	1	1.38 (1.03–1.84)	-	-	2	1.50 (1.05–2.14)	0.0%	0.52
No RT at baseline	7	1.11 (1.03–1.20)	0.0%	0.49	6	2.22 (1.52–3.24)	60.6%	0.03	8	1.13 (0.95–1.35)	5.6%	0.39
High-quality studies^c^	11	1.13 (1.03–1.25)	31.5%	0.15	5	1.77 (1.23–2.54)	70.3%	0.009	14	1.11 (0.92–1.32)	23.7%	0.20
Analyses except												
Outlier study^d^	13	1.13 (1.01–1.26)	40.2%	0.07	5	1.55 (1.15–2.08)	37.9%	0.17	17	1.11 (0.97–1.28)	11.1%	0.32
Calculated RRs^e^	12	1.12 (1.01–1.24)	38.3%	0.09	7	1.96 (1.38–2.80)	66.2%	0.007	16	1.11 (0.96–1.28)	16.3%	0.27
	All-cause mortality				Stroke				HF			
	*N* studies	RR (95% CI)	*I* ^2^	*P* value^a^	*N* studies	RR (95% CI)	*I* ^2^	*P* value^a^	*N* studies	RR (95% CI)	*I* ^2^	*P* value^a^
Statistical model												
Random effects	40	1.25 (1.13–1.39)	86.9%	<0.001	9	1.09 (0.96–1.24)	52.3%	0.03	8	1.13 (0.98–1.30)	54.8%	0.03
Fixed effects	40	1.20 (1.16–1.23)	-	-	9	1.03 (0.97–1.09)	-	-	8	1.07 (1.00–1.14)	-	-
Analyses with												
Large cohort^b^	7	1.18 (0.94–1.48)	97.0%	<0.001	2	1.01 (0.86–1.19)	86.3%	0.007	2	1.03 (0.95–1.11)	0.0%	0.87
No RT at baseline	20	1.32 (1.10–1.59)	92.0%	<0.001	1	0.93 (0.85–1.01)	-	-	4	1.02 (0.95–1.09)	0.0%	0.90
High-quality studies^c^	34	1.25 (1.11–1.39)	88.6%	<0.001	9	1.09 (0.96–1.24)	52.3%	0.03	6	1.17 (0.99–1.39)	66.3%	0.01
Analyses except												
Outlier study^d^	32	1.30 (1.22–1.39)	26.4%	0.09	8	1.12 (1.04–1.21)	0.0%	0.43	7	1.07 (0.94–1.21)	34.4%	0.16
Calculated RRs^e^	38	1.27 (1.15–1.41)	87.1%	<0.001	9	1.09 (0.96–1.24)	52.3%	0.03	8	1.13 (0.98–1.30)	54.8%	0.03
	MI				AF				Total cardiovascular events			
	*N* studies	RR (95% CI)	*I* ^2^	*P* value^a^	*N* studies	RR (95% CI)	*I* ^2^	*P* value^a^	*N* studies	RR (95% CI)	*I* ^2^	*P* value^a^
Statistical model												
Random effects	7	1.15 (1.05–1.25)	0.0%	0.43	4	1.02 (0.71–1.46)	70.6%	0.02	8	1.16 (0.97–1.38)	73.4%	<0.001
Fixed effects	7	1.15 (1.05–1.25)	-	-	4	1.02 (0.85–1.21)	-	-	8	1.06 (1.01–1.11)	-	-
Analyses with												
Large cohort^b^	2	1.12 (1.02–1.24)	0.0%	0.55	2	1.09 (0.59–2.02)	89.9%	0.002	2	1.15 (0.84–1.57)	92.2%	<0.001
No RT at baseline	4	1.12 (1.02–1.23)	0.0%	0.57	1	0.80 (0.62–1.03)	-	-	3	1.12 (0.80–1.56)	25.2%	0.26
High-quality studies^c^	7	1.15 (1.05–1.25)	0.0%	0.43	3	1.06 (0.66–1.70)	79.9%	0.007	8	1.19 (0.99–1.43)	73.5%	<0.001
Analyses except												
Outlier study^d^	7	1.15 (1.05–1.25)	0.0%	0.43	3	0.83 (0.67–1.03)	0.0%	0.85	7	1.28 (1.16–1.40)	0.0%	0.50
Calculated RRs^e^	6	1.14 (1.05–1.24)	0.0%	0.58	4	1.02 (0.71–1.46)	70.6%	0.02	8	1.16 (0.97–1.38)	73.4%	<0.001

*AF* atrial fibrillation, *CI* confidence interval, *HF* heart failure, *IHD* ischemic heart disease, *MI* myocardial infarction, *RR* relative risk, *RT* replacement therapy

^a^ *P* value for heterogeneity

^b^Large cohort studies with >10,000 population

^c^High-quality studies with a total score of ≥6

^d^Outlier studies found by sensitivity analyses (hetred command); there were 2 for cardiac mortality, 8 for all-cause mortality, 1 for stroke, 1 for HF, 1 for AF, and 1 for total cardiovascular events

^e^Three cohort studies did not report the estimates: Aho et al. [21] (cardiovascular mortality), Bai et al. [20] (IHD, all-cause mortality, MI), Parle et al. [19] (all-cause mortality)

A priori subgroup analyses were conducted across key study characteristics (Table 2); higher risk of IHD associated with hypothyroidism was consistently observed in some of these analyses. The estimates did not differ significantly between population-based and convenience samples studies. There was no significantly higher risk of IHD events in cardiac patients with hypothyroidism. However, this should be considered seriously for only three studies included. The estimates also did not differ significantly across other subgroups. In additional subgroup analyses by TSH levels in patients with SCH compared with euthyroidism, we observed a significantly higher risk of IHD in SCH with TSH level ≥10.0 mIU/L (RR: 1.32; 95% CI: 1.00–1.74; Additional file 2: Table S4). Univariate meta-regressions showed that these a priori factors did not affect the overall effects (Table 3).

**Table 2:** Subgroup and heterogeneity analyses of pooled RRs of primary outcomes

Factors	IHD					Cardiac mortality				
	*N* studies	Events/participants	RR (95% CI)	*I* ^2^	*P* value^a^	*N* studies	Events/participants	RR (95% CI)	*I* ^2^	*P* value^a^
Degrees of hypothyroidism										
All hypothyroidism	4	13,444/593,499	1.13 (1.04–1.23)	0.0%	Reference	2	282/2836	1.83 (0.72–4.62)	90.0%	0.75
SCH	8	3400/21,623	1.12 (0.89–1.42)	55.8%	0.74	5	819/32,086	2.00 (1.34–2.97)	47.8%	Reference
OHypo	1	18/474	2.54 (0.87–7.41)	-	0.30	-	-	-	-	-
Study population										
Convenience sample	4	1406/8946	1.08 (0.66–1.79)	58.2%	0.73	5	398/6595	2.33 (1.39–3.91)	70.7%	Reference
Cardiac patients^b^	3	1277/8149	1.15 (0.68–1.94)	67.7%	-	4	393/5596	2.22 (1.28–3.83)	76.5%	-
Population-based	9	15,456/606,650	1.12 (1.00–1.25)	36.8%	Reference	2	703/28,327	1.43 (1.10–1.86)	0.0%	0.33
Mean age										
≥80 years	-	-	-	-		-	-	-	-	-
65–79 years	8	3417/20,298	1.06 (0.89–1.27)	37.4%	0.45	2	236/3077	1.91 (0.61–5.93)	82.3%	0.68
<65 years	5	13,445/595,298	1.19 (1.02–1.39)	51.0%	Reference	5	865/31,845	2.07 (1.39–3.07)	61.8%	Reference
Location										
Europe	7	13,907/600,542	1.10 (0.95–1.26)	31.7%	0.23	4	814/31,087	1.91 (1.28–2.86)	52.8%	0.26
North America	4	2675/12,727	1.08 (0.95–1.24)	15.2%	0.22	1	205/2430	1.14 (0.76–1.71)	-	Reference
Asia	1	18/474	2.54 (0.87–7.41)	-	Reference	2	82/1405	3.02 (2.00–4.56)	0.0%	0.10
Oceania	1	262/1853	1.80 (1.20–2.70)	-	0.63	-	-	-	-	-
Female proportion										
≥50%	9	15,773/610,042	1.07 (0.98–1.18)	19.9%	0.08	3	708/29,326	1.47 (1.12–1.94)	2.3%	Reference
<50%	4	1089/5554	1.47 (1.04–2.07)	40.9%	Reference	4	393/5596	2.22 (1.28–3.83)	76.5%	0.52
Mean follow-up										
≥10 years	5	3615/45,383	1.18 (0.98–1.42)	55.4%	Reference	3	708/29,326	1.47 (1.12–1.94)	2.3%	Reference
5–10 years	2	11,491/557,482	1.13 (1.02–1.25)	0.0%	0.73	-	-	-	-	-
<5 years	6	1756/12731	1.07 (0.75–1.53)	53.9%	0.59	4	393/5596	2.22 (1.28–3.83)	76.5%	0.52
Factors	Cardiovascular mortality					All-cause mortality				
	*N* studies	Events/participants	RR (95% CI)	*I* ^2^	*P* value^a^	*N* studies	Events/participants	RR (95% CI)	*I* ^2^	*P* value^a^
Degrees of hypothyroidism										
All hypothyroidism	5	2724/20,707	0.99 (0.70–1.39)	42.0%	0.35	21	98,621^c^/876,811	1.21 (1.07–1.37)	87.6%	0.51
SCH	12	2549/137,310	1.15 (1.00–1.32)	0.0%	Reference	18	13,529/159,104	1.32 (1.13–1.54)	65.9%	Reference
OHypo	-	-	-	-	-	1	6/474	1.78 (0.21–14.98)	-	0.80
Study population										
Convenience sample	5	1454/12,229	1.15 (0.95–1.39)	0.0%	0.70	20	9004^c^/54,577	1.45 (1.27–1.66)	63.4%	0.009
Cardiac patients^b^	2	1333/9820	1.10 (0.83–1.45)	0.0%	-	13	4643^c^/38,219	1.51 (1.26–1.81)	65.7%	-
Population-based	12	3819/145,788	1.08 (0.86–1.36)	33.3%	Reference	20	103,152/981,812	1.10 (0.95–1.28)	91.7%	Reference
Mean age										
≥80 years	1	87/539	0.55 (0.33–0.93)	-	0.004	3	6216/18,267	1.05 (0.55–1.98)	95.2%	Reference
65–79 years	11	2799/22,989	1.08 (0.94–1.23)	0.0%	0.04	20	9706/49,057	1.15 (1.03–1.29)	34.7%	0.73
<65 years	5	2387/134,489	1.53 (1.16–2.01)	0.0%	Reference	17	96,234^c^/969,065	1.38 (1.19–1.60)	89.3%	0.22
Location										
Europe	7	1807/13,446	0.96 (0.74–1.23)	33.4%	0.045	19	92,477/847,659	1.09 (0.95–1.26)	83.2%	0.92
North America	5	2544/24,417	1.08 (0.90–1.29)	0.0%	0.097	10	8176^c^/40,891	1.26 (1.12–1.41)	54.5%	0.50
Asia	4	731/118,301	1.72 (1.13–2.62)	0.0%	Reference	10	10,671/143,981	1.75 (1.42–2.15)	60.1%	0.07
Oceania	1	191/1853	1.50 (0.90–2.50)	-	0.70	1	832/3858	1.06 (0.86–1.31)	-	Reference
Female proportion										
≥50%	12	3814/149,254	1.06 (0.87–1.29)	29.5%	Reference	22	103,550/990,510	1.13 (0.97–1.31)	91.1%	Reference
<50%	5	1459/8763	1.23 (0.96–1.58)	0.0%	0.35	18	8606^c^/45,879	1.42 (1.25–1.63)	65.8%	0.03
Mean follow-up										
≥10 years	7	3338/139,867	1.21 (0.98–1.50)	16.2%	0.12	9	14,896/165,332	1.25 (0.98–1.59)	89.7%	0.44
5–10 years	5	399/4517	1.19 (0.94–1.52)	0.0%	0.20	13	88,149^c^/822,913	1.10 (0.92–1.32)	85.8%	Reference
<5 years	5	1536/13,633	0.88 (0.63–1.23)	34.0%	Reference	18	9111^c^/48,144	1.37 (1.19–1.58)	72.7%	0.09

*CI* confidence interval, *IHD* ischemic heart disease, *OHypo* overt hypothyroidism, *RR* relative risk, *SCH* subclinical hypothyroidism

^a^P values indicate whether the pooled estimate in each subgroup differs from a nominated reference subgroup

^b^Studies with cardiac patients were included in convenience sample studies

^c^Two cohort studies by McQuade et al. [45] and Mitchell et al. [46] did not provide the number of all-cause mortality events; we contacted the authors by email but didn’t get a reply

**Table 3:** Univariate meta-regression of hypothyroidism on IHD events and mortality

Variable	IHD				Cardiovascular mortality				All-cause mortality			
	*N* studies	Scale	Exp(*b*)^a^ (95% CI)	*P* value^b^	*N* studies	Scale	Exp(*b*)^a^ (95% CI)	*P* value^b^	*N* studies	Scale	Exp(b)^a^ (95% CI)	*P* value^b^
Degrees of hypothyroidism												
SCH	8	Per unit	0.97 (0.77, 1.22)	0.74	12	Per unit	Reference	-	18	Per unit	Reference	-
OHypo	1		2.25 (0.44, 11.36)	0.30	0		-	-	1		1.36 (0.12, 15.24)	0.80
All	4		Reference	-	5		0.86 (0.61, 1.20)	0.35	21		0.93 (0.73, 1.17)	0.51
Study population	13	Per unit	1.05 (0.77, 1.43)	0.73	17	Per unit	1.06 (0.75, 1.50)	0.70	40	Per unit	1.32 (1.08, 1.62)	0.009
Mean age												
≥80 years	0	Per unit	-	-	1	Per unit	0.36 (0.19, 0.69)	0.004	3	Per unit	Reference	-
65–79 years	8		0.91 (0.70, 1.19)	0.45	11		0.70 (0.50, 0.98)	0.04	20		1.07 (0.72, 1.59)	0.73
<65 years	5		Reference	-	5		Reference	-	17		1.27 (0.86, 1.87)	0.22
Location												
Europe	7	Per unit	0.44 (0.10, 1.86)	0.23	7	Per unit	0.57 (0.33, 0.99)	0.045	19	Per unit	1.03 (0.62, 1.71)	0.92
North America	4		0.43 (0.10, 1.83)	0.22	5		0.63 (0.36, 1.10)	0.097	10		1.19 (0.71, 2.01)	0.50
Oceania	1		0.71 (0.15, 3.32)	0.63	1		0.87 (0.40, 1.90)	0.70	1		Reference	-
Asia	1		Reference	-	4		Reference	-	10		1.65 (0.96, 2.82)	0.07
Female proportion	13	Per 1%	0.79 (0.35, 1.75)	0.53	17	Per 1%	0.64 (0.25, 1.64)	0.33	40	Per 1%	0.71 (0.39, 1.29)	0.25
Mean follow-up years	13	Per 1 year	1.02 (0.99, 1.05)	0.17	17	Per 1 year	1.03 (0.99, 1.06)	0.096	40	Per 1 year	0.99 (0.97, 1.02)	0.53

*CI* confidence interval, *IHD* ischemic heart disease, *OHypo* overt hypothyroidism, *SCH* subclinical hypothyroidism

^a^Exp (*b*) is the exponential of the regression coefficient and describes how the outcome variable changes with a unit increase in the explanatory variable

^b^ *P* value for Exp (*b*)

### Hypothyroidism and cardiac mortality

Of seven studies analyzed with cardiac mortality, there were 1101 events among 34,922 participants. All studies adjusted for age and sex, six adjusted for smoking, less than half adjusted for other main cardiovascular risk factors, and only one study adjusted for RT.

**Fig. 3:**
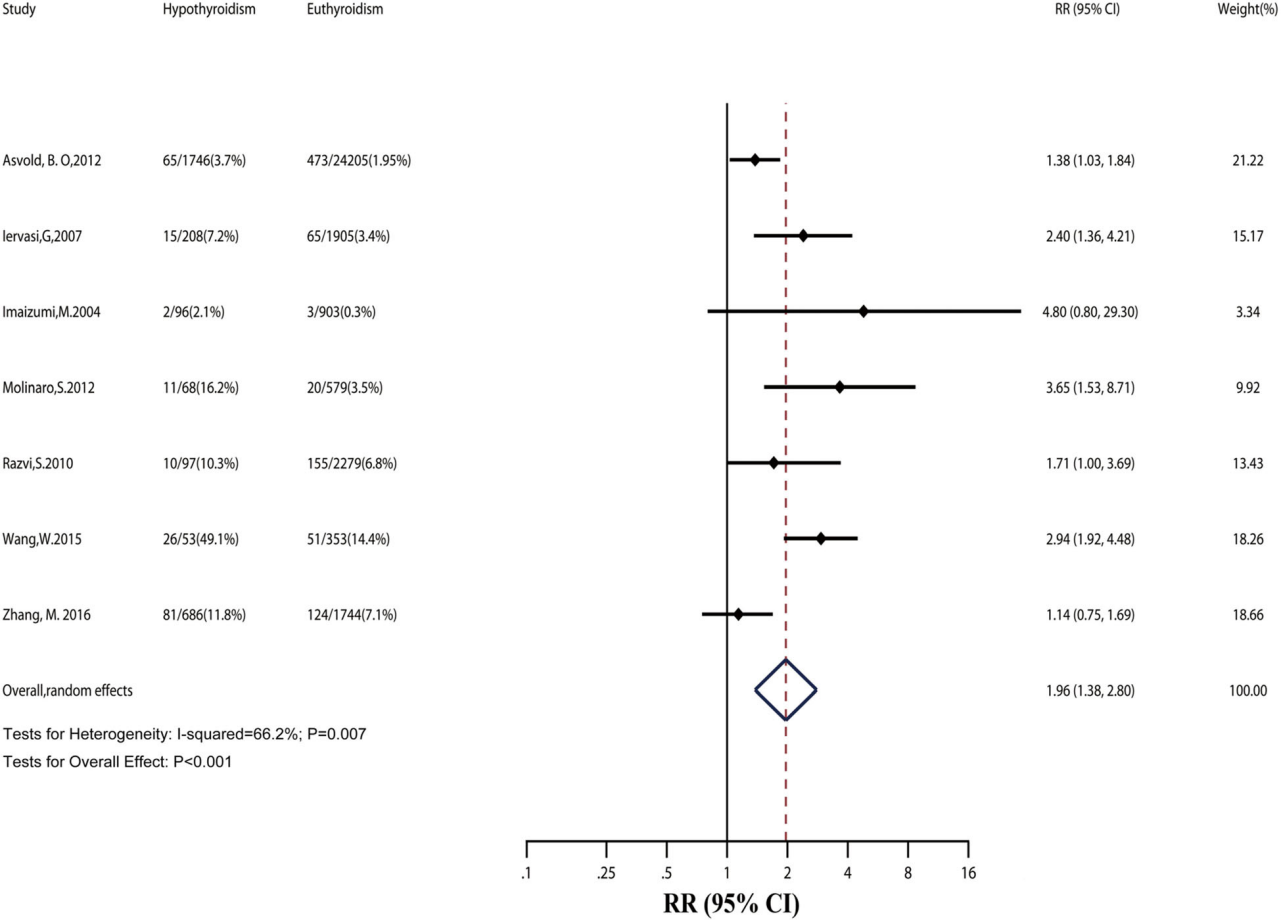
Relative risk (*RR*) of cardiac mortality associated with hypothyroidism compared with euthyroidism. The *dotted line* in forest plot represents pooled RR value in random effects model. *CI* confidence interval

Evidence synthesis for cardiac mortality showed a statistically strong association with hypothyroidism (RR: 1.96; 95% CI: 1.38–2.80; Fig. 3), with moderate heterogeneity (*I* ^2^: 66.2%; *P* = 0.007). Sensitivity analysis by hetred command in Stata found that two outlier studies might be the source of heterogeneity [33, 34]. When the two outlier studies were removed, there was evidence of low heterogeneity in the five remaining studies (*I* ^2^: 37.9%; *P* = 0.17; Table 1). The heterogeneity may have been due to the smaller sample size of the outlier studies than that of the other five studies (Fig. 3).

Subgroup analyses did not find the source of heterogeneity. However, we found that the risk of cardiac mortality was also significantly higher in SCH (Table 2). Besides, we observed that cardiac patients with hypothyroidism might have a higher risk of cardiac mortality than cardiac patients with euthyroidism (RR: 2.22; 95% CI: 1.28–3.83). Sensitivity analyses with large cohorts or high-quality studies obtained similar results. Considering only seven studies in this section, we did not conduct meta-regression and funnel plots.

### Hypothyroidism and cardiovascular mortality

Seventeen studies were included for cardiovascular mortality, involving 158,017 participants and 5273 events. Fourteen studies adjusted for age, thirteen adjusted for sex, more than half adjusted for cardiovascular risk factor, and only four adjusted for RT.

**Fig. 4:**
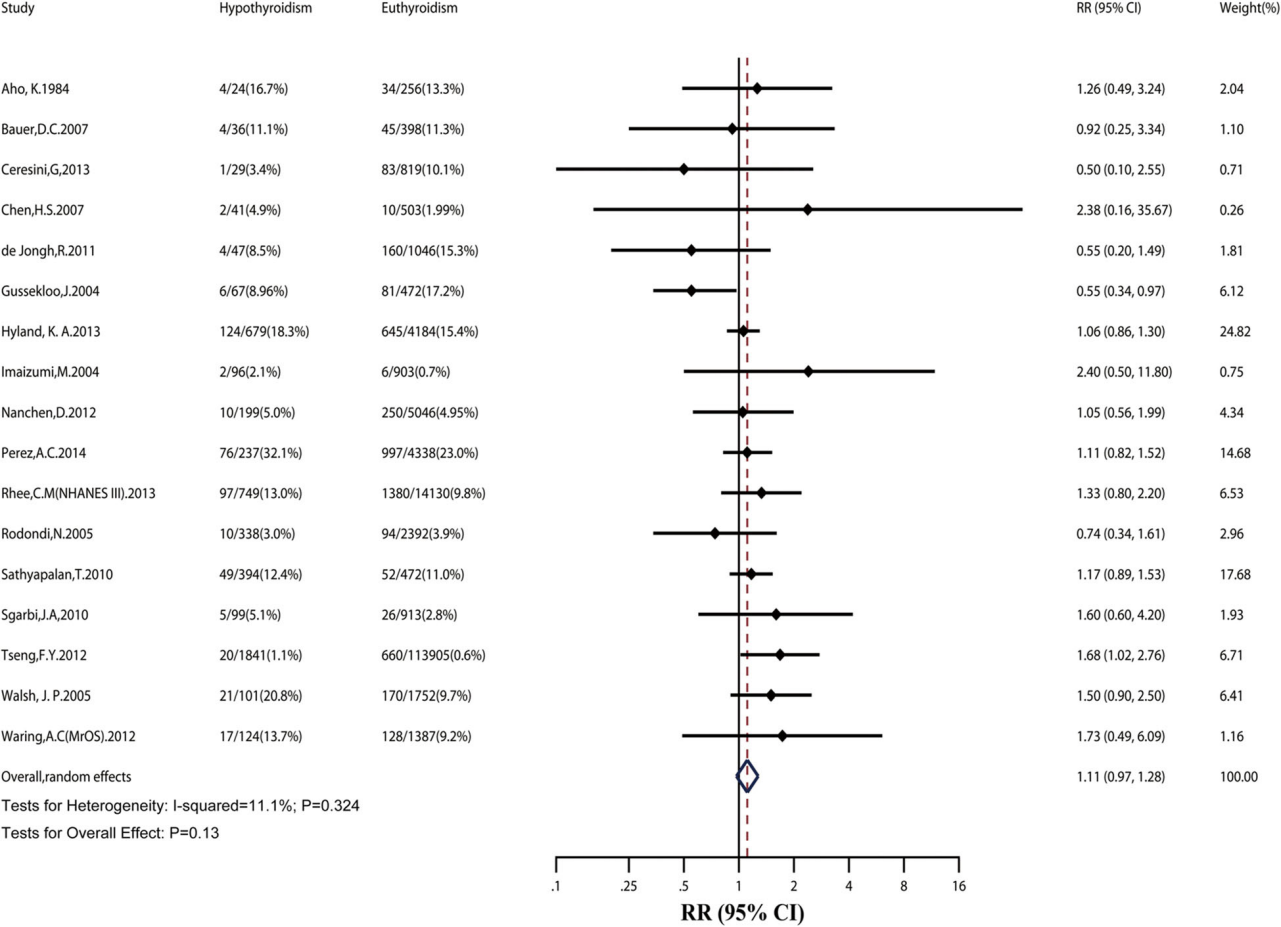
Relative risk (*RR*) of cardiovascular mortality associated with hypothyroidism compared with euthyroidism. The *dotted line* in forest plot represents pooled RR value in random effects model. *CI* confidence interval

The summary RR of cardiovascular mortality associated with hypothyroidism was 1.11 (95% CI: 0.97–1.28; Fig. 4), with low heterogeneity (*I* ^2^: 11.1%; *P* = 0.324). In sensitivity analyses, the risk estimates did not change after analyzing with studies removing RT at baseline or with high-quality studies. No publication bias was found (Egger test: *P* = 0.92). In meta-regression and subgroup analyses, the pooled RRs of each group or subgroup were almost similar to the total.

### Hypothyroidism and all-cause mortality

All-cause mortality occurred in at least 112,156 patients among 1,036,389 participants from 40 studies. Thirty-five studies adjusted for age, thirty-one adjusted for sex, most adjusted for cardiovascular risk factors, and only five adjusted for RT.

**Fig. 5:**
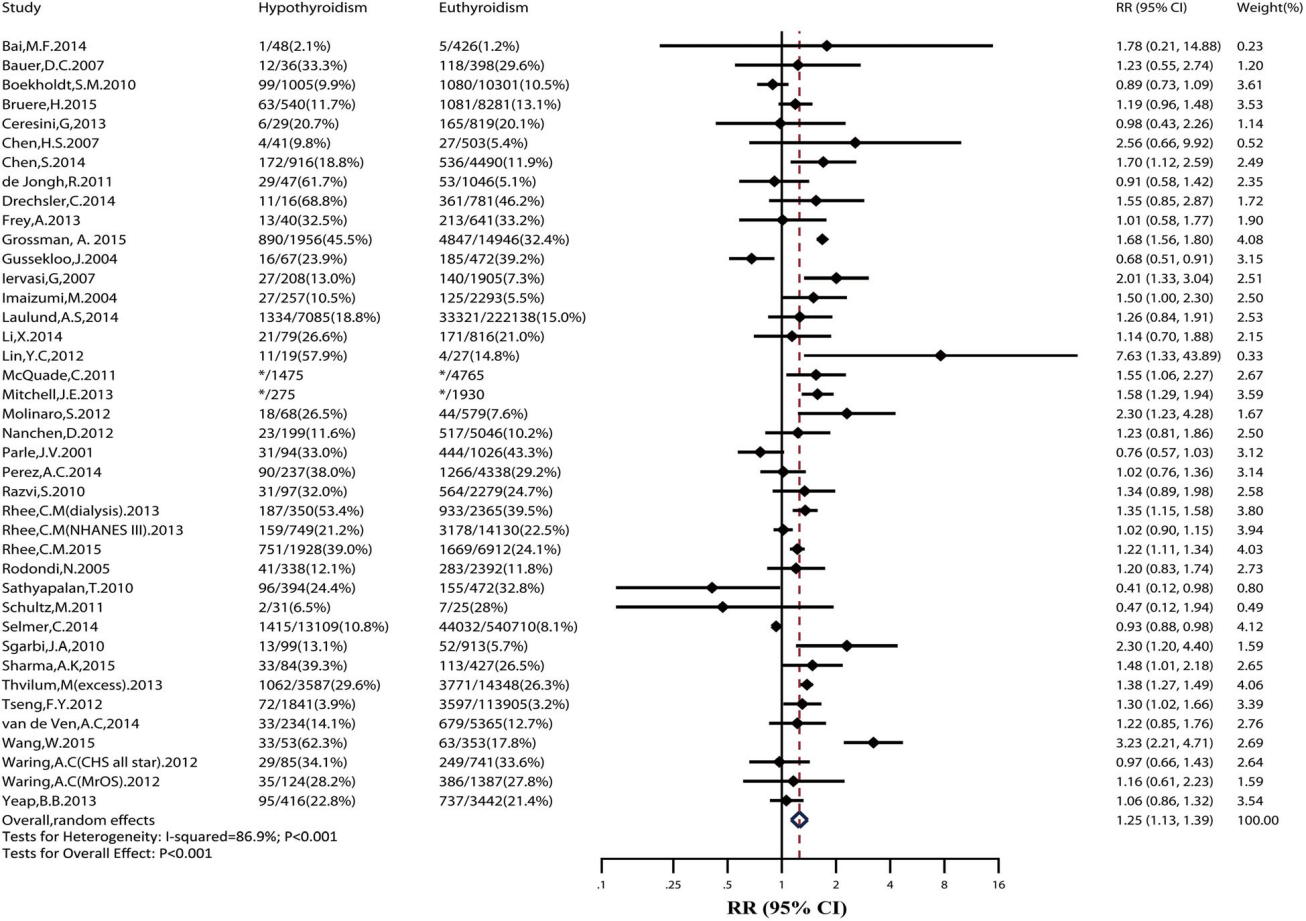
Relative risk (*RR*) of all-cause mortality associated with hypothyroidism compared with euthyroidism. *Two cohort studies by McQuade et al. [45] and Mitchell et al. [46] did not provide the number of all-cause mortality events; we contacted the authors by email but didn’t receive a reply. The *dotted line* in forest plot represents pooled RR value in random effects model. *CI* confidence interval

The pooled RR value of all-cause mortality was 1.25 (95% CI: 1.13–1.39; Fig. 5), with high heterogeneity (*I* ^2^: 86.9%; *P* < 0.001). In subgroup and meta-regression analyses, we observed evidence for heterogeneity by study population (*P* = 0.009). The RR for all-cause mortality in convenience samples was significantly higher than that in population-based studies; also, the risk of all-cause mortality in cardiac patients with hypothyroidism was significantly higher than that in cardiac patients with euthyroidism (RR: 1.51; 95% CI: 1.26–1.81). We observed that SCH was also associated with a higher risk of all-cause mortality (RR: 1.32; 95% CI: 1.13–1.54).

In sensitivity analyses, the pooled RR value changed little after inclusion of large cohorts, studies without RT at baseline, or high-quality studies. Sensitivity analyses by hetred command in Stata found eight outlier studies [16, 18, 19, 29, 33, 35–37]. When they were removed, the pooled RRs of the remaining studies were still significant (RR: 1.30; 95% CI: 1.22–1.39; *I* ^2^ = 26.4%). No publication bias was found (Egger test: *P* = 0.44).

### Hypothyroidism and stroke, HF, MI, AF, and total cardiovascular events

**Fig. 6:**
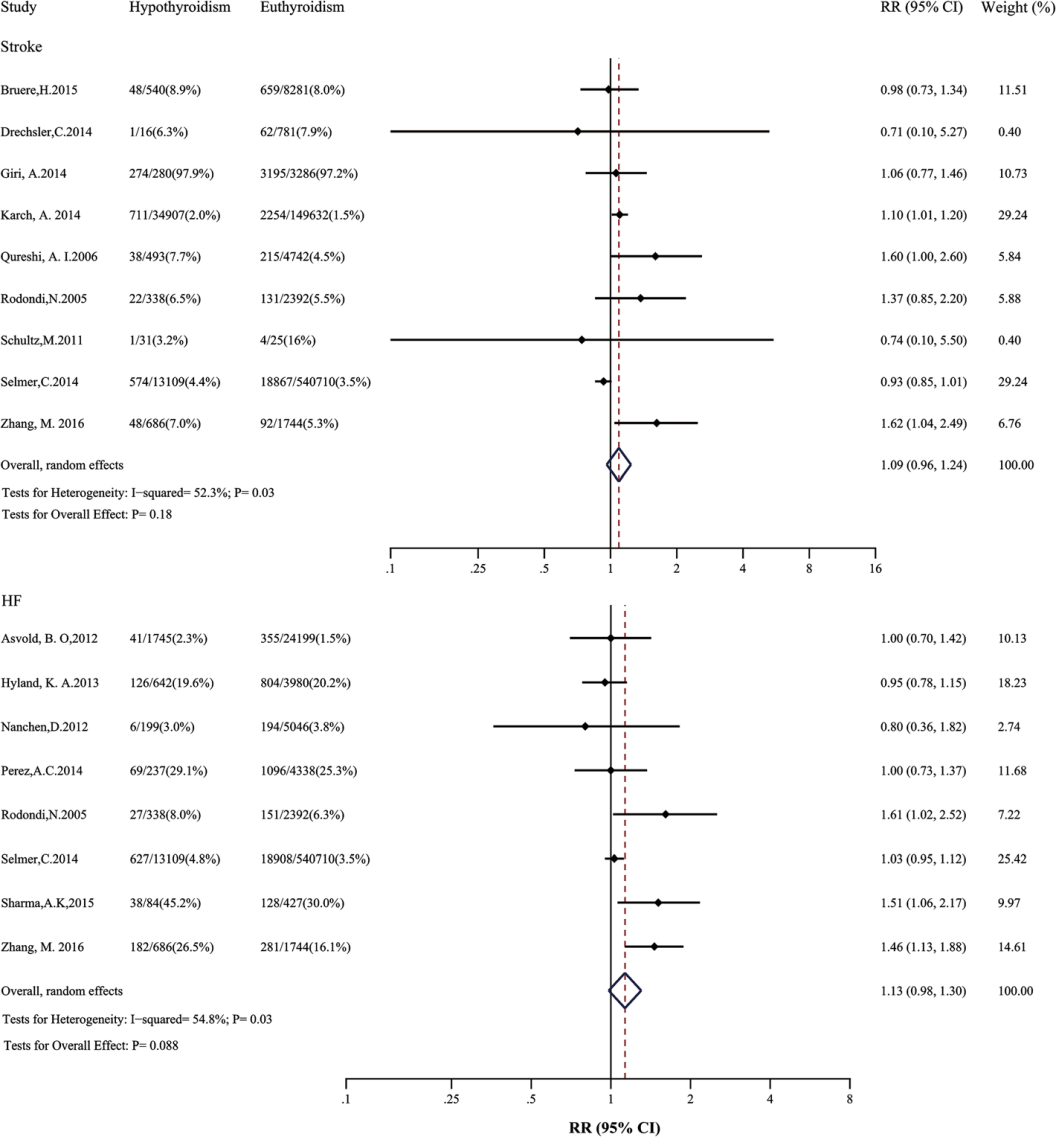
Relative risk (*RR*) of stroke and heart failure (*HF*) associated with hypothyroidism compared with euthyroidism. The *dotted line* in forest plot represents pooled RR value in random effects model. *CI* confidence interval

For stroke, nine studies were included, reporting 27,196 events among 761,993 participants. All studies adjusted for age, eight adjusted for sex, most adjusted for cardiovascular risk factors, and only one adjusted for RT. The overall RR of stroke was 1.09 (95% CI: 0.96–1.24; Fig. 6) with moderate heterogeneity (*I* ^2^: 52.3%; *P* = 0.03). After excluding one outlier study [16] with population >10,000, RR changed slightly with no heterogeneity (*I* ^2^: 0.0%; *P* = 0.43).

Of eight studies analyzed with HF, there were 23,033 events among 599,876 participants. Seven studies adjusted for age, all adjusted for sex, most adjusted for cardiovascular risk factors, and only two adjusted for RT. Evidence synthesis for HF did not show a significant association with hypothyroidism (RR: 1.13; 95% CI: 0.98–1.30; Fig. 6), with moderate heterogeneity across studies (*I* ^2^: 54.8%; *P* = 0.03). After excluding one outlier study [5], the heterogeneity decreased with the pooled RR still insignificant. We performed an additional subgroup analysis of risk of HF by mean age (Additional file 2: Table S5). The risk estimates did not significantly change in each subgroup.

**Fig. 7:**
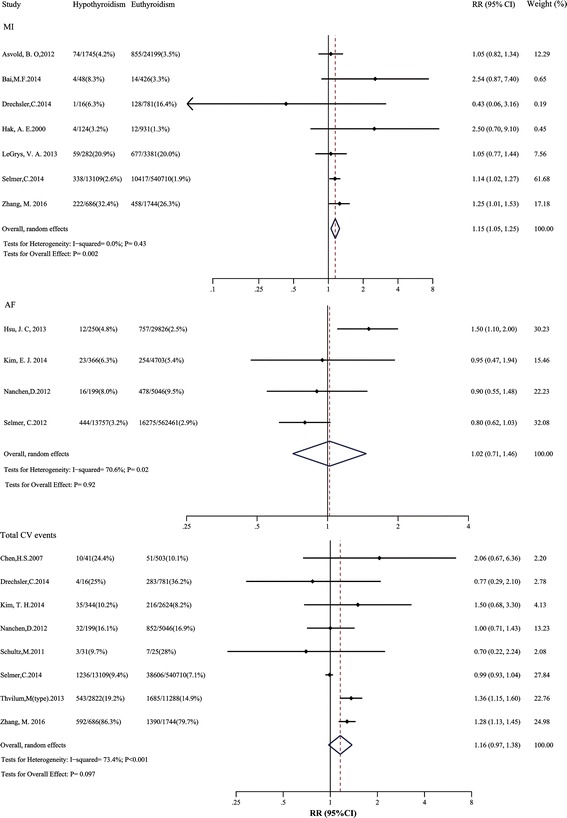
Relative risks of MI, AF and total cardiovascular events in hypothyroidism compared with euthyroidism. The *dotted line* in forest plot represents pooled RR value in random effects model. *AF* atrial fibrillation; *CI *confidence interval; *CV* cardiovascular; *MI* myocardial infarction; *RR* Relative risks

Seven studies involving 588,182 participants were analyzed reporting a total of 13,263 MI. Six studies adjusted for age, four adjusted for sex, most adjusted for cardiovascular risk factors, and none adjusted for RT. Hypothyroidism was significantly associated with higher risk of MI (RR: 1.15; 95% CI: 1.05–1.25; Fig. 7), with no heterogeneity (*I* ^2^: 0.0%; *P* = 0.43). The same results were obtained after using fixed-effect models, inclusion of large cohorts, studies without RT at baseline, or high-quality studies.

For AF, four studies were included, reporting 18,259 events among 616,608 individuals. All studies adjusted for age and sex, most adjusted for cardiovascular risk factors, and only one adjusted for RT. The pooled RR value of AF was 1.02 (95% CI: 0.71–1.46; Fig. 7), with moderate heterogeneity (*I* ^2^: 70.6%; *P* = 0.02). After excluding one outlier study [38] of HIV-infected patients with a large population size, no heterogeneity was found (*I* ^2^: 0.0%; *P* = 0.85), but the RR was still not statistically significant.

Eight studies involving 579,969 participants were analyzed reporting a total of 45,545 total cardiovascular events. Six studies adjusted for age, six adjusted for sex, most adjusted for cardiovascular risk factors, and none adjusted for RT. The pooled RR for total cardiovascular events was 1.16 (95% CI: 0.97–1.38; Fig. 7), with moderate heterogeneity (*I* ^2^: 73.4%; *P* < 0.001). Similar results were observed in the sensitivity analyses.

## Discussion

In this analysis of 1,898,314 participants from 55 cohort studies, hypothyroidism is associated with higher risks of IHD, MI, cardiac mortality, and all-cause mortality. These associations mostly persist in sensitivity analyses, indicating the stability of our results. In subgroup analyses, we also observe that SCH is significantly associated with higher risks of cardiac mortality (Table 2) and IHD (TSH level ≥10.0 mIU/L; Additional file 2: Table S4). Moreover, cardiac patients with hypothyroidism, compared with those with euthyroidism, have a significantly higher risk of cardiac mortality and all-cause mortality.

Our study is the first meta-analysis to evaluate risks of cardiovascular events and mortality in hypothyroidism with no restriction of hypothyroid states and study population. In subgroup analyses, our results are generally consistent with previous meta-analyses showing modest higher risks of IHD and cardiac mortality associated with SCH or SCH with TSH levels ≥10.0 mIU/L [7–9, 39]. However, previous meta-analyses included limited studies, and had a wider 95% CI than ours, reflecting less statistical power. Our separate analysis with MI still shows a significant higher risk in hypothyroidism, which further supports the higher risk of IHD. We observe that cardiac patients with hypothyroidism experience a significantly higher risk of cardiac mortality, but not IHD or MI, than cardiac patients with euthyroidism. There may be two reasons for this. First, small numbers of studies may weaken the reliability of the results. Second, effects of hypothyroidism on IHD events and MI might differ in different cardiac diseases, which require further prospective studies to prove.

Previous meta-analyses show conflicting results of risk of all-cause mortality in SCH. Some [8–10], but not all [7, 39], meta-analyses find a higher risk of all-cause mortality. Including 40 studies in all-cause mortality analyses, we find that hypothyroidism is associated with a higher risk of all-cause mortality in both the general public and patients with cardiac disease.

However, our findings could not indicate that hypothyroidism is an independent risk factor. RT might be one of the important confounders, for only a small number of cohort studies adjusted for RT (Additional file 2: Table S3). Future prospective studies should pay more attention to the RT information of each participant and adjust the results for as many confounders as possible.

We find no significant association between hypothyroidism and risks of stroke, HF, or AF. One previous IPD meta-analysis focused on risk of stroke in SCH [40]. The authors found that there was a higher risk of stroke in SCH for people younger than 65 years, but no overall effect of SCH on stroke could be demonstrated. The other IPD meta-analysis found that risks of HF events were increased with a TSH level of 10.0–19.9 mIU/L [41]. Similarly, our meta-analyses results found no overall effect of all hypothyroidism on stroke and HF. However, as stroke and HF events were secondary outcomes, we did not further conduct the subgroup analyses.

Higher risks of IHD, MI, and cardiac mortality with hypothyroidism might be associated with the known effects of thyroid hormone on the circulatory system. Altered endothelial function, increased atherosclerosis, and altered coagulability have been reported to be associated with hypothyroidism and may accelerate the development of IHD [42].

A retrospective cohort study of patients from the United Kingdom General Practitioner Research Database found that levothyroxine therapy in SCH was associated with fewer IHD events in younger individuals [43]. However, this study is observational and susceptible to residual confounding [44]. More research is needed to understand the effects of treatment of SCH.

### Strengths and limitations

The strengths of this meta-analysis include strict inclusion criteria, many more cohort studies included than ever before, and predefined sensitivity and subgroup analyses. There is no publication bias, which favors stability of the findings. Moreover, we do not restrict hypothyroid states and study population, thus realizing a full-scale assessment of the effects of hypothyroidism.

There are some limitations. Firstly, heterogeneity is observed in some outcomes. However, we have found all sources of heterogeneity, and sensitivity analyses confirm the stability of our results. Secondly, the absence of IPD might influence the accuracy of the results. Thirdly, thyroid function testing was mostly performed at baseline, which would weaken the accuracy of the results regarding the possible change of thyroid function during follow-up. Another limitation is the different TSH values used across studies to define thyroid disease.

## Conclusions

Our analyses suggest that hypothyroidism or SCH is a risk factor for IHD and cardiac mortality. Moreover, hypothyroidism is associated with higher risks of cardiac mortality and all-cause mortality compared with euthyroidism in both the general public and cardiac patients. Our analyses offer more reliable evidence of higher risks of IHD events and mortality in patients with hypothyroidism, and highlight that cardiac patients with hypothyroidism should be considered seriously for higher risk of cardiac mortality and all-cause mortality. However, more high-quality prospective cohort studies and randomized controlled trials are needed to draw firmer conclusions.

## Additional files

Additional file 1: The PRISMA checklist. (DOC 841 kb)
Additional file 2: Summary of included cohort studies, quality assessment, the adjusting variables in each cohort study, additional subgroup analyses by TSH levels in SCH, subgroup analysis of risk of HF by mean age, and funnel plots. (DOC 63 kb)

## Abbreviations

AF: Atrial fibrillation
BMI: Body mass index
CI: Confidence interval
FT4: Free tetraiodothyronine
HF: Heart failure
HR: Hazard ratio
IHD: Ischemic heart disease
IPD: Individual participant data
IRR: Incidence rate ratio
MI: Myocardial infarction
OHypo: Overt hypothyroidism
OR: Odds ratio
RR: Relative risk
RT: Replacement therapy
SCH: Subclinical hypothyroidism
TSH: Thyroid-stimulating hormone
