# Supplementary material for: What is the association of hypothyroidism with risks of cardiovascular events and mortality? A meta-analysis of 55 cohort studies involving 1,898,314 participants
Source: BMC Med. 2017 Feb 2;15:21. doi: 10.1186/s12916-017-0777-9 (PMC5289009; doi:10.1186/s12916-017-0777-9)
Supplement: Additional file 1: — The PRISMA checklist. (DOC 841 kb) [file 12916_2017_777_MOESM1_ESM.doc]

**Additional file 1**

**What is the association of hypothyroidism with risks of cardiovascular events and mortality: a meta- analysis of 55 cohort studies involving 1,898,314 participants**

**Content**

**Table S1:** Summary of included cohort studies in the present meta-analysis

**Table S2:** Quality Assessment of Included Cohort Studies Using the Newcastle-Ottawa Scale

**Table S3:** The adjusting variables in each cohort study included in the meta-analysis

**Table S4:** Additional subgroup analyses of main outcomes by TSH levels in SCH

**Table S5:** Subgroup analysis of risk of heart failure by mean age

**Figure S1:** Funnel plots for publication bias of included cohort studies

**Supplementary References**

| **Table S1. Summary of included cohort studies in the present meta-analysis** | | | | | | | | | | |
| --- | --- | --- | --- | --- | --- | --- | --- | --- | --- | --- |
| **Study** | **Country** | **Study type** | **Study population** | **No.** | **Female (%)** | **Mean age (y)** | **Mean follow-up (y)** | **Exclusion of patients with RT at baseline** | **Degrees of hypothyroidism** | **Outcomes (risk estimates)** |
| **Aho K et al. 1984** | Finland | Prospective cohort | Men from rural areas of eastern and southwestern Finland | 280 | 0 | 64.5 | 5 | No | All hypothyroidism | Cardiovascular mortality (calc) |
| **Asvold BO et al. 2012** | Norway | Prospective cohort | Population-based study  (the HUNT study) | 25951 | 67.7 | 53 | 12 | Yes | SCH: TSH 3.6-4.0 mIU/L; or TSH>4 mIU/L, FT4>8 pmol/l;  OHypo: TSH>4 mIU/L, FT4 < 8.0 pmol/l | AMI (HR)  HF (HR)  Cardiac mortality (HR) |
| **Bai MF et al. 2014** | China | Prospective cohort | Suspected CHD patients admitted for CAG | 474 | 32.4 | 64.9 | 1.3 | No | OHypo: TSH>4.78 mIU/L, FT3/FT4 ↓ | All-cause mortality (calc)  MI (calc) |
| **Bauer DC et al.2007** | USA | Prospective cohort | Population-based study; ( the SOF) | 434 | 100 | 72.6 | 11.9 | No | All hypothyroidism: TSH>5.5 mIU/L | Cardiovascular mortality (HR)  All-cause mortality (HR) |
| **Boekholdt et al. 2010** | UK | Prospective cohort | Population-based study  (The EPIC study) | 11306 | 54.9 | 58 | 10.6 | Yes | SCH: TSH>4 mIU/L, FT4 N;  OHypo: TSH>4 mIU/L, FT4 ↓; | IHD (HR)  All-cause mortality (HR) |
| **Bruere H et al. 2015** | France | Retrospective cohort | AF patients | 8821 | 38.7 | 71 | 2.5 | No | All hypothyroidism | All-cause mortality (HR)  Stroke (HR) |
| **Ceresini G et al. 2013** | Italy | Prospective cohort | Population-based study  (the InCHIANTI study) | 848 | 57 | 75.5 | 6 | Yes | SCH: TSH>4.68 mIU/L, FT4 N | Cardiovascular mortality (HR)  All-cause mortality (HR) |
| **Chen HS et al. 2007** | China, Taiwan | Prospective cohort | Type 2 diabetic patients | 544 | 35.1 | 66.4 | 3.67 | Yes | SCH: 4 mIU/L <TSH<20 mIU/L, FT4 N | CVD (HR)  Cardiovascular mortality (HR)  All-cause mortality (HR) |
| **Chen S et al. 2014** | Israel | Retrospective cohort | HF patients;  Data from Health Maintenance Organization | 5406 | 51.2 | 75 | 1.2 | No | All hypothyroidism  (TSH 4.5mIU/L) | All-cause mortality (HR) |
| **De Jongh R et al. 2011** | Netherlands | Prospective cohort | Population-based study;  the Longitudinal Aging Study Amsterdam (LASA) | 1093 | 50.5 | 75.5 | 10.7 | Yes | SCH: TSH>4.5 mIU/L, T3/4 N | Cardiovascular mortality (HR)  All-cause mortality (HR) |
| **Drechsler C et al. 2014** | German | Prospective cohort | Type 2 diabetes mellitus, and undergoing hemodialysis for less than 2 years (4D study) | 797 | 46.3 | 65.6 | 4 | No | SCH: TSH 4.1-15 mIU/L, FT3/4 N | MI (HR)  Stroke (HR)  All-cause mortality (HR)  MACCE (HR) |
| **Frey A et al. 2013** | Germany | Prospective cohort | Patients hospitalized for systolic HF (LVEF≤ 40%) (Interdisciplinary Network Heart Failure study, INH study) | 681 | 29 | 68 | 3.08 | No | SCH: TSH>4 mIU/L, FT3/4 N; | All-cause mortality (HR) |
| **Giri A et al. 2014** | USA | Prospective cohort | Postmenopausal women  (the WHI-OS study) | 3566 | 100 | 67.5 | 7 | No | SCH: TSH≥4.69 mIU/L, FT4 N; | Ischemic stroke (HR) |
| **Grossman A et al.2016** | Israel | Retrospective cohort | Population-based study  (CHMO database) | 16902 | 59 | 83 | 10 | Yes | SCH: TSH>4.2 mIU/L, FT4 N | All-cause mortality (HR) |
| **Gussekloo et al 2004** | Netherlands | Prospective cohort | The Leiden 85-Plus Study | 539 | 66 | 85 | 3.7 | No | SCH: TSH>4.8 mIU/L, FT4 N  OHypo: TSH>4.8 mIU/L, FT4↓； | All-cause mortality (HR)  Cardiovascular mortality (HR) |
| **Hak AE et al. 2000** | Netherlands | Prospective cohort | The Rotterdam Study in 1990 to 1993 | 1055 | 100 | 69.0 | 4.6 | Yes | SCH: TSH>4 mIU/L, FT4 N | MI (RR) |
| **Hsu JC et al. 2013** | USA | Retrospective cohort | HIV-infected veterans | 30076 | 2.77 | 50.25 | 6.8 | No | All hypothyroidism | AF (HR) |
| **Hyland KA et al.2013** | USA | Prospective cohort | Population-based study  (Cardiovascular Health Study, CHS) | 4863 | 56 | 74 | 10 | Yes | SCH: TSH 4.5-20 mIU/L, FT4 N | IHD (HR)  Cardiovascular mortality (HR)  HF (HR) |
| **Iervasi G et al 2007** | Italy | Prospective cohort | Cardiac patients | 2113 | 32.6 | 61.1 | 2.7 | Yes | SCH: TSH 4.5-10 mIU/L,  FT3/FT4 N. | Cardiac mortality (HR)  All-cause mortality (HR) |
| **Imaizumi M et al. 2004** | Japan | Prospective cohort | Atomic bomb survivors | 2550 | 60.8 | 58.5 | 12.2 | Yes | SCH: TSH>5 mIU/L, FT4 N | IHD mortality (HR)  Cardiovascular mortality (HR)  All-cause mortality (HR) |
| **Karch A et al. 2014** | UK | Retrospective cohort | AIT patients versus no AIT patients; data from The Health Improvement Network (THIN) database | 184539 | 80.3 | 58 | 3 | No | All hypothyroidism | Stroke (RR) |
| **Kim EJ et al.2014** | USA | Prospective cohort | Population-based study  (The Framingham Heart Study) | 5069 | 52 | 57 | 10 | No | All hypothyroidism: TSH 4.5-19.9; | AF (HR) |
| **Kim TH et al. 2014** | Korea | Prospective cohort | Population-based study  (Ansung cohort study) | 2968 | 54.9 | 55 | 10 | Yes | SCH:TSH≥4.1 mIU/L, FT4 N | CVD (HR) |
| **Laulund AS et al. 2014** | Denmark | Retrospective cohort | Population-based study | 229196 | 56.3 | 51.3 | 7.4 | Yes | SCH: TSH>4 mIU/L, T4 N;  OHypo: TSH>4 mIU/L, T4↓ | All-cause mortality (HR) |
| **LeGrys VA et al 2013** | USA | Prospective cohort | Postmenopausal women  (WHI-OS) | 3663 | 100 | 67.5 | 7 | Yes | SCH: TSH>4.68 mIU/L, FT4 N | Incident MI (HR) |
| **Li X et al. 2014** | China | Prospective cohort | Dilated cardiomyopathy Patients | 895 | 26.3 | 52.1 | 3.5 | No | SCH: TSH>5.5 mIU/L, FT4 N | All-cause mortality (HR) |
| **Lin YC et al. 2012** | China  Taiwan | Retrospective cohort | Long-term(>8y) peritoneal dialysis patients, | 46 | 47.8 | 44 | 25 | Yes | All hypothyroidism  (SCH: TSH>4.0mIU/L, FT4 N; OHypo: TSH>4.0mIU/L, FT4↓) | All-cause mortality (HR) |
| **McQuadeC et al. 2011** | USA | Retrospective cohort | CHD patients or patients with management of CHD risk factors  (PreCIS database) | 6240 | 42.1 | 55.2 | 8 | No | SCH-mild: 3.1–6.0 mIU/L;  SCH-moderate: 6.1–10 mIU/L; hypo: >10 mIU/L | All-cause mortality (HR) |
| **Mitchell JE et al. 2013** | USA  Canada  New Zealand | Prospective cohort | Ischemic or nonischemic NYHA functional class II or III HF and LVEF ≤35% (SCD-HeFT) | 2205 | 24.1 | 59.5 | 3.8 | No | All hypothyroidism: TSH>5 mIU/L | All-cause mortality (HR) |
| **Molinaro S et al. 2012** | Italy | Prospective cohort | Acute patients admitted  to the Emergency Cardiac Care of the National Research  Council Institute of Clinical Physiology in Pisa | 647 | 29.7 | 67.7 | 2.5 | Yes | SCH: TSH 4.5-10 mIU/L, FT3/4 N; | Cardiac mortality (HR)  All-cause mortality (HR) |
| **Nanchen D et al. 2012** | Netherlands, Scotland, Ireland | Prospective cohort | Patients with known cardiovascular  risk factors or previous CVD (The Prospective Study of Pravastatin in the Elderly at Risk, PROSPER) | 5245 | 50.5 | 75 | 3.2 | No | SCH: TSH≥4.5 mIU/L, FT4 N | IHD, CVD, HF, AF (HR)  Cardiovascular mortality (HR)  All-cause mortality (HR) |
| **Parle JV et al. 2001** | UK | Prospective cohort | Population-based study | 1120 | 57.2 | 70.4 | 8.2 | Yes | All hypothyroidism: TSH>0.5 mIU/L | All-cause mortality (calc) |
| **Perez AC et al. 2014** | Scotland Norway  UK, Sweden Netherlands | Prospective cohort | Symptomatic (NYHA class II to IV), ischemic,  systolic (LVEF≤40%) HF(no more than 35% in patients in NYHA class II) (the CORONA study) | 4575 | 22.3 | 72.6 | 2.7 | Yes | Hypo: TSH>5 mIU/L | Cardiovascular mortality (HR)  All-cause mortality (HR)  HF hospitalizations (HR) |
| **Qureshi AI et al. 2006** | USA | Prospective cohort | Population-based study  (NHANES I, NHEFS) | 5235 | 54.8 | 48 | 20 | No | All hypothyroidism: FT4 index<3.5 (FT4 index: the product of T4 and resin T3 uptake) | Stroke (RR) |
| **Razvi S et al. 2010** | UK | Prospective cohort | Population-based study  ( the Whickham Survey) | 2376 | 52.9 | 45.5 | 20 | Yes | SCH: TSH 6-15 mIU/L, TT4 N | IHD (HR)  IHD mortality (HR)  All-cause mortality (HR) |
| **Rhee CM (****dialysis). 2013** | USA | Retrospective cohort | Dialysis patients  (data from the Partners Healthcare Research Patient Data Repository, RPDR) | 2715 | 44.2 | 63.3 | 1.7 | No | All hypothyroidism: TSH>5 mIU/L | All-cause mortality (HR) |
| **Rhee CM (NHANES III). 2013** | USA | Retrospective cohort | Population-based study  (NHANES III) | 14879 | 53 | 46.1 | 14.3 | No | SCH: TSH≥4.6 mIU/L, FT4 N;  OHypo: TSH≥4.6 mIU/L, FT4 N↓ | Cardiovascular Mortality (HR)  All-cause mortality (HR) |
| **Rhee CM et al. 2015** | USA | Retrospective cohort | Hemodialysis patients  (data from a large US dialysis organization, LDO) | 8840 | 49 | 65 | 1 | No | All hypothyroidism: TSH>5 mIU/L | All-cause mortality (HR) |
| **Rodondi N et al.2005** | USA | Prospective cohort | healthy community-dwelling men and women  (the Health, Aging, and Body Composition Study) | 2730 | 51 | 74.7 | 4 | No | SCH: TSH≥4.5 mIU/L, T4 N | IHD (HR)  Cardiovascular mortality (HR)  All-cause mortality (HR)  HF (HR)  Stroke (HR) |
| **SathyapalanT et al.2010** | UK | Retrospective cohort | Type 2 diabetes  (a diabetes database at Hull Royal Infirmary） | 866 | 50.8 | 72.1 | 7.9 | No | SCH: TSH>4.67 mIU/L, FT4 N | Cardiovascular mortality (OR)  All-cause mortality (OR) |
| **Schultz M et al.2011** | Denmark | Prospective cohort | Population-based study | 56 | 58.2 | 67.9 | 5 | No | SCH: TSH>4 mIU/L, FT3/4 N | All-cause mortality (HR)  MACCE (HR)  Stroke (HR) |
| **Selmer C et al. 2012** | Denmark | Retrospective cohort | Population-based study | 576218 | 61 | 48.9 | 5.5 | Yes | SCH: TSH>5 mIU/L, FT4/TT4 N  OHypo: TSH>5 mIU/L, FT4/TT4↓ | AF (IRR) |
| **Selmer C et al. 2014** | Denmark | Retrospective cohort | Population-based study (citizens of Copenhagen who underwent thyroid function testing at the Copenhagen General Practitioners Laboratory) | 553819 | 61 | 48.6 | 5.5 | Yes | SCH: TSH>5 mIU/L, FT3/4 N  OHypo: TSH>5 mIU/L, FT3/4↓ | All-cause mortality (IRR)  MI (IRR)  HF (IRR)  Stroke (IRR)  MACCE (IRR) |
| **Sgarbi JA et al. 2010** | Brazil | Prospective cohort | Japanese–Brazilians  (the Japanese-Brazilian thyroid study) | 1012 | 53.2 | 56.9 | 7.5 | Yes | SCH: TSH>4.5 mIU/L, FT4 N; | Cardiovascular mortality (HR)  All-cause mortality (HR) |
| **Sharma AK et al. 2015** | USA | Retrospective cohort | HF patients receiving CRT | 511 | 22.7 | 68.5 | 3 | No | All hypothyroidism (TSH≥5.0 mIU/L) | All-cause mortality (HR)  HF hospitalizations (HR) |
| **Thvilum M (type). 2013** | Denmark | Retrospective cohort | Population-based study | 14110 | 84 | 58 | 6 | No | All hypothyroidism | CVD (HR) |
| **Thvilum. (excess)**  **2013** | Denmark | Retrospective cohort | 5% sample from the Danish Background Population DCRS/DDB | 17935 | 84.4 | 60 | 5.6 | No | All hypothyroidism | All-cause mortality (HR) |
| **Tseng FY et al. 2012** | China,  Taiwan | Retrospective cohort | Data were collected from 4 private nationwide MJ Health Screening Centers in Taiwan | 115746 | 52.5 | 43 | 10 | Yes | SCH: TSH 5-19.96 mIU/L, TT4 N; | Cardiovascular mortality (RR)  All-cause mortality (RR) |
| **van de Ven AC et al. 2014** | Netherlands | Prospective cohort | Population-based study;  Nijmegen Biomedical Study | 5599 | 52.5 | 55.7 | 9.4 | Yes | SCH: TSH>4 mIU/L, FT4 N；  OHypo: TSH>4 mIU/L, FT4↓ | All-cause mortality (HR) |
| **Walsh JP et al. 2005** | Australia | Prospective cohort | Population-based study  (Busselton Health Study) | 1853 | 49.6 | 49.8 | 20 | No | SCH: TSH>4 mIU/L, FT4 N | IHD (HR)  Cardiovascular mortality (HR) |
| **Wang W et al. 2015** | China | Prospective cohort | Idiopathic Dilated Cardiomyopathy (the National Center of Cardiovascular diseases) | 406 | 29 | 51 | 1.4 | Yes | SCH: TSH>5.78 mIU/L, FT3/4 N;  OHypo: TSH>4.78 mIU/L, FT3/4↓ | Cardiac mortality (HR)  All-cause mortality (HR) |
| **Waring (MrOS). 2012** | USA | Prospective cohort | Population-based study  (The MrOS study) | 1587 | 0 | 74 | 8.3 | No | SCH: TSH≥4.79 mIU/L, FT4 N | Cardiovascular mortality (HR)  All-cause mortality (HR) |
| **Waring AC (CHS All Stars) 2012** | USA | Prospective cohort | The CHS All Stars Study in 2005–2006 | 826 | 60.9 | 85.3 | 5.1 | Yes | SCH: TSH 4.50–19.99 mIU/L, FT4 N | All-cause mortality (HR) |
| **Yeap BB et al. 2013** | Australia | Prospective cohort | Population-based study | 3858 | 0 | 77 | 6.4 | Yes | SCH: TSH>4 mIU/L, FT4 N | All-cause mortality (HR) |
| **Zhang M et al. 2016** | USA | Retrospective cohort | Patients who undergo PCI at Mayo Clinic 1994-2009 | 2430 | 36 | 64.6 | 3.0 | No | All hypothyroidism: TSH>5.0 mIU/L | Cardiac mortality (HR)  MI (HR)  HF (HR)  Stroke (HR)  MACCE (HR) |

AF, atrial fibrillation; AIT, autoimmune thyroiditis; AMI, acute myocardial infarction; CAG, coronary angiography; CHD, coronary heart disease; CRT, cardiac resynchronization therapy; CVD, cardiovascular disease; FT3, free triiodothyronine; FT4, free thyroxine; HF, heart failure; HIV, human immunodeficiency virus; HR, hazard ratio; IHD, ischemia heart disease; IRR, incidence rate ratio; LVEF, left ventricular ejection fraction; MACCE, major adverse cardiac cerebral events; MI, myocardial infarction; N, normal; NYHA, New York Heart Association; OHypo, overt hypothyroidism; OR, odds ratio; PCI, percutaneous coronary intervention; RR, relative risk; SCH, subclinical hypothyroidism; TSH, thyroid-stimulating hormone.

| **Table S2. Quality Assessment of Included Cohort Studies Using the Newcastle-Ottawa Scale** | | | | | | | | | | | | |
| --- | --- | --- | --- | --- | --- | --- | --- | --- | --- | --- | --- | --- |
| **Author** | **Selection** | | | |  | **Comparability** | | **`** | **Outcome** | | | **Total Quality**  **Score** |
|  | **Representative-**  **ness of Exposed**  **Cohort** | **Selection of Non-Exposed Cohort** | **Ascertainment Of Exposure** | **Demonstration That Outcome of Interest Was Not Present at Start of Study** |  | **Adjust for age** | **Adjust for cardiovascular risk factors** |  | **Assessment of outcome** | **Follow-up**  **length** | **Loss to follow-up rate** |  |
| Aho K, 1984 | 1 | 1 | 1 | 0 |  | 0 | 0 |  | 1 | 1 | 1 | 6 |
| Asvold BO, 2012 | 1 | 1 | 1 | 1 |  | 1 | 1 |  | 1 | 1 | 1 | 9 |
| Bai MF, 2014 | 0 | 1 | 1 | 0 |  | 0 | 0 |  | 1 | 0 | 1 | 4 |
| Bauer DC, 2007 | 1 | 1 | 1 | 1 |  | 1 | 1 |  | 1 | 1 | 1 | 8 |
| Boekholdt SM, 2010 | 1 | 1 | 1 | 1 |  | 1 | 1 |  | 1 | 1 | 0 | 8 |
| Bruere H，2015 | 0 | 1 | 1 | 1 |  | 1 | 1 |  | 1 | 0 | 0 | 6 |
| Ceresini G, 2013 | 1 | 1 | 1 | 0 |  | 1 | 1 |  | 1 | 1 | 0 | 7 |
| Chen HS, 2007 | 0 | 1 | 1 | 1 |  | 1 | 1 |  | 1 | 0 | 1 | 7 |
| Chen S, 2014 | 0 | 1 | 1 | 0 |  | 1 | 1 |  | 1 | 0 | 1 | 6 |
| de Jongh R, 2011 | 1 | 1 | 1 | 1 |  | 1 | 1 |  | 1 | 1 | 0 | 8 |
| Drechsler C, 2014 | 0 | 1 | 1 | 0 |  | 1 | 1 |  | 1 | 0 | 1 | 6 |
| Frey A, 2013 | 0 | 1 | 1 | 0 |  | 1 | 0 |  | 1 | 0 | 1 | 5 |
| Giri A, 2014 | 1 | 1 | 1 | 1 |  | 1 | 1 |  | 1 | 1 | 0 | 8 |
| Grossman A, 2015 | 1 | 1 | 1 | 1 |  | 1 | 1 |  | 1 | 1 | 0 | 8 |
| Gussekloo J, 2004 | 1 | 1 | 1 | 1 |  | 1 | 1 |  | 1 | 0 | 1 | 8 |
| Hak AE, 2000 | 1 | 1 | 1 | 1 |  | 1 | 1 |  | 1 | 0 | 1 | 8 |
| Hsu JC, 2013 | 0 | 1 | 1 | 1 |  | 1 | 1 |  | 1 | 1 | 0 | 7 |
| Hyland KA, 2013 | 1 | 1 | 1 | 1 |  | 1 | 0 |  | 1 | 1 | 0 | 7 |
| Iervasi G, 2007 | 0 | 1 | 1 | 0 |  | 1 | 1 |  | 1 | 0 | 1 | 6 |
| Imaizumi M, 2004 | 0 | 1 | 1 | 0 |  | 1 | 0 |  | 1 | 1 | 0 | 5 |
| Karch A, 2014 | 0 | 1 | 1 | 1 |  | 1 | 1 |  | 1 | 0 | 1 | 7 |
| Kim EJ, 2014 | 1 | 1 | 1 | 1 |  | 1 | 1 |  | 1 | 1 | 1 | 9 |
| Kim TH, 2014 | 1 | 1 | 1 | 1 |  | 1 | 1 |  | 1 | 1 | 0 | 8 |
| Laulund AS, 2014 | 1 | 1 | 1 | 0 |  | 1 | 1 |  | 1 | 1 | 1 | 8 |
| LeGrys VA, 2013 | 1 | 1 | 1 | 1 |  | 1 | 0 |  | 1 | 1 | 1 | 8 |
| Li X, 2014 | 0 | 1 | 1 | 0 |  | 1 | 1 |  | 1 | 0 | 1 | 6 |
| Lin YC, 2012 | 0 | 1 | 1 | 0 |  | 1 | 1 |  | 1 | 1 | 1 | 7 |
| McQuade C, 2011 | 0 | 1 | 1 | 0 |  | 1 | 1 |  | 1 | 1 | 1 | 7 |
| Mitchell JE, 2013 | 0 | 1 | 1 | 0 |  | 1 | 1 |  | 1 | 0 | 1 | 6 |
| Molinaro S, 2012 | 0 | 1 | 1 | 0 |  | 1 | 1 |  | 1 | 0 | 0 | 5 |
| Nanchen D, 2012 | 0 | 1 | 1 | 0 |  | 1 | 1 |  | 1 | 0 | 0 | 5 |
| Parle JV, 2001 | 1 | 1 | 1 | 1 |  | 1 | 0 |  | 1 | 1 | 0 | 7 |
| Perez AC, 2014 | 0 | 1 | 1 | 0 |  | 1 | 1 |  | 1 | 0 | 0 | 5 |
| Qureshi AI, 2006 | 1 | 1 | 1 | 1 |  | 1 | 1 |  | 1 | 1 | 1 | 9 |
| Razvi S, 2010 | 1 | 1 | 1 | 1 |  | 1 | 1 |  | 1 | 1 | 1 | 9 |
| Rhee CM (dialysis), 2013 | 0 | 1 | 1 | 0 |  | 1 | 1 |  | 1 | 0 | 1 | 6 |
| Rhee CM (NHANES III), 2013 | 1 | 1 | 1 | 0 |  | 1 | 1 |  | 1 | 1 | 1 | 8 |
| Rhee CM, 2015 | 0 | 1 | 1 | 0 |  | 1 | 1 |  | 1 | 0 | 1 | 6 |
| Rodondi N, 2005 | 1 | 1 | 1 | 0 |  | 1 | 1 |  | 1 | 0 | 1 | 7 |
| Sathyapala T, 2010 | 0 | 1 | 1 | 0 |  | 1 | 1 |  | 1 | 1 | 0 | 6 |
| Schultz M, 2011 | 1 | 1 | 1 | 0 |  | 1 | 1 |  | 1 | 1 | 1 | 8 |
| Selmer C, 2012 | 1 | 1 | 1 | 1 |  | 1 | 1 |  | 1 | 1 | 1 | 9 |
| Selmer C, 2014 | 1 | 1 | 1 | 1 |  | 1 | 0 |  | 1 | 1 | 1 | 8 |
| Sgarbi JA, 2010 | 1 | 1 | 1 | 0 |  | 1 | 1 |  | 1 | 1 | 1 | 8 |
| Sharma AK, 2015 | 0 | 1 | 1 | 0 |  | 1 | 1 |  | 1 | 0 | 1 | 6 |
| Thvilum M (excess), 2013 | 1 | 1 | 1 | 0 |  | 1 | 1 |  | 1 | 1 | 1 | 8 |
| Thvilum M (type), 2013 | 1 | 1 | 1 | 0 |  | 1 | 1 |  | 1 | 1 | 1 | 8 |
| Tseng FY, 2012 | 1 | 1 | 1 | 0 |  | 1 | 1 |  | 1 | 1 | 1 | 8 |
| van de Ven AC, 2014 | 1 | 1 | 1 | 0 |  | 1 | 1 |  | 1 | 1 | 1 | 8 |
| Walsh JP, 2005 | 1 | 1 | 1 | 1 |  | 1 | 1 |  | 1 | 1 | 1 | 9 |
| Wang W, 2015 | 0 | 1 | 1 | 0 |  | 1 | 1 |  | 1 | 0 | 1 | 6 |
| Waring AC (CHS all star), 2012 | 1 | 1 | 1 | 0 |  | 1 | 0 |  | 1 | 1 | 0 | 6 |
| Waring AC (MrOS), 2012 | 1 | 1 | 1 | 0 |  | 1 | 1 |  | 1 | 1 | 1 | 8 |
| Yeap BB, 2013 | 1 | 1 | 1 | 0 |  | 1 | 1 |  | 1 | 1 | 1 | 8 |
| Zhang M, 2016 | 0 | 1 | 1 | 0 |  | 1 | 1 |  | 1 | 0 | 1 | 6 |

We used the Newcastle-Ottawa scale to evaluate the quality of cohort studies. A study can be awarded a maximum of one score for each numbered item within the Selection and Outcome categories and a maximum of two scores for Comparability.

**Selection**:

1) Representativeness of exposed cohort: 1, truly or somewhat representative of a community/ population-based study; 0, selected group of users, eg population from hospital patients or patients with a kind of disease, or no description of the derivation of the cohort.

2) Selection of non-exposed cohort: 1, drawn from the same community as the exposed cohort; 0, drawn from a different source or no description of the derivation of the non-exposed cohort.

3) Ascertainment of exposure: 1, Validation of hypothyroidism with secure record or structured interview; 0, written self-report or no description.

4) Demonstration that outcome of interest was not present at start of study: 1, yes; 0, no.

**Comparability**:

1) 1, study adjusted for age; 1, study adjusted for other cardiovascular risk factors.

**Outcome**:

1) Assessment of outcome: 1, confirmed by medical records or record linkage; 0, self-reported or no description.

2) Was follow-up long enough for outcomes to occur: 1, duration of follow-up >= 5 years; 0, if duration of follow-up < 5 years.

3) Loss to follow-up rate: 1, complete follow-up or loss to follow up rate <=20 %; 0, follow-up rate < 80% or no statement

| **Table S3. The adjusting variables in each cohort study included in the meta-analysis** | | | | | | | | | |
| --- | --- | --- | --- | --- | --- | --- | --- | --- | --- |
| **Study** | **Age** | **Sex** | **BMI/ weight/ waist-hip ratio/ waist circumference** | **Smoking** | **Cholesterol** | **Hypertension/ blood pressure** | **Diabetes/ HbA1c/ glucose** | **L-T4 Replacement therapy** | **Other variables** |
| **Aho K et al. 1984** |  |  |  |  |  |  |  |  |  |
| **Asvold BO et al. 2012** | √ | √ | √ | √ |  |  |  |  |  |
| **Bai MF et al. 2014** |  |  |  |  |  |  |  |  |  |
| **Bauer DC et al.2007** | √ | All females | √ |  |  |  |  | √ | Baseline use of estrogen |
| **Boekholdt et al. 2010** | √ | √ | √ | √ | √ | √ | √ |  |  |
| **Bruere H et al. 2015** | √ | √ |  |  |  | √ | √ |  | Chronic HF, previous Stroke, vascular disease, abnormal renal/ liver function, stroke, bleeding history or predisposition, labile international normalized ratio, drugs/ alcohol |
| **Ceresini G et al. 2013** | √ | √ | √ | √ |  |  |  |  | Log IL-6, TNF-alpha, educational level, depression, CHD (including angina and myocardial infarction), chronic HF, stroke and cancer |
| **Chen HS et al. 2007** | √ | √ | √ | √ | √ | √ | √ |  | Medication, urinary albumin: creatinine excretion |
| **Chen S et al. 2014** | √ | √ | √ |  | √ | √ | √ | √ | IHD, AF, pulse, serum urea levels, glomerular filtration rate, hemoglobin, serum sodium, ACEI/ARB, beta-blocker, furosemide, spironolactone, thiazide, digoxin, amiodarone, aspirin |
| **De Jongh R et al. 2011** | √ | √ | √ | √ | √ | √ |  |  | Alcohol use, physical activity, number of chronic diseases, heart rate |
| **Drechsler C et al. 2014** | √ | √ | √ |  |  | √ |  |  | Atorvastatin, left ventricular hypertrophy, albumin level, creatinine level, NT-proBNP level, and ultrafiltration volume |
| **Frey A et al. 2013** | √ |  |  |  |  |  |  |  |  |
| **Giri A et al. 2014** | √ | All females | √ | √ |  | √ | √ |  | Ethnicity, gravidity, hormone therapy, alcohol consumption, physical activity, years since menopause |
| **Grossman A et al.2016** | √ | √ |  | √ |  | √ | √ |  | CRF, COPD, dementia, CVA, chronic HF |
| **Gussekloo^a^ et al 2004** | √ | √ |  |  |  |  |  |  | Baseline disability, levels of albumin and CRP, Mini-Mental State  Examination score, number of chronic diseases, subjective health |
| **Hak AE et al. 2000** | √ | All females | √ | √ | √ | √ |  |  |  |
| **Hsu JC et al. 2013** | √ | √ | √ | √ |  | √ | √ |  | Race, time-updated CD4+ cell count, HIV RNA viral load, CAD, congestive heart failure, chronic lung disease, alcoholism, estimated glomerular filtration rate, proteinuria |
| **Hyland KA et al.2013** | √ | √ |  |  |  |  |  | √ |  |
| **Iervasi G et al 2007** | √ | √ |  |  |  |  |  |  | Ischemic and non-ischemic heart disease, TSH, FT3, FT4 |
| **Imaizumi M et al. 2004** | √ | √ |  | √ |  |  |  |  |  |
| **Karch A et al. 2014** | √ | √ | √ | √ | √ | √ | √ |  | Alcohol, AF, CHD, congestive HF |
| **Kim EJ et al.2014** | √ | √ | √ | √ |  |  |  | √ | Hypertension treatment, significant cardiac murmur, prevalent CVD, PR interval |
| **Kim TH et al. 2014** |  |  |  |  |  |  |  |  | Atherosclerotic cardiovascular disease risk score |
| **Laulund AS et al. 2014** | √ | √ |  |  |  |  |  |  | Charlson Comorbidity Index, location of TSH collection |
| **LeGrys VA et al 2013** | √ | All females |  | √ |  |  |  |  | Ethnicity, gravidity, hormone therapy, alcohol consumption |
| **Li X et al. 2014** | √ | √ |  | √ |  | √ |  |  | AF, ventricular tachycardia, drinking status, disease duration, QRS duration, left ventricular diameter, left atrial diameter, LVEF, FT3, T3, T4, subclinical hyperthyroidism, subclinical hypothyroidism, NT-proBNP levels, and amiodarone medicine |
| **Lin YC et al. 2012** |  |  |  |  |  |  | √ |  | Cardiothoracic ratio, CRP, CVD |
| **McQuadeC et al. 2011** | √ |  | √ | √ |  | √ | √ |  | Primary versus secondary prevention categorization, history of cerebrovascular disease, the log transformation of homocysteine and triglycerides |
| **Mitchell JE et al. 2013** | √ | √ |  |  |  | √ | √ |  | HF etiology, NYHA class, mitral regurgitation, renal  insufficiency, substance abuse, LVEF, time since HF diagnosis, electrocardiographic measures, 6-min walk distance, Duke Activity Status Index, ACEI or digoxin use |
| **Molinaro S et al. 2012** | √ | √ |  | √ |  |  |  |  | CAD, history of percutaneous angioplasty, CABG, LVEF, CRP, creatinine and triglycerides |
| **Nanchen D et al. 2012** | √ | √ | √ | √ | √ | √ | √ |  | Education, CVD, creatinine, and use of β-blockers and antiarrhythmic |
| **Parle JV et al. 2001** |  |  |  |  |  |  |  |  |  |
| **Perez AC et al. 2014** | √ | √ | √ | √ |  | √ | √ |  | NYHA class, LVEF, heart rate, MI, angina pectoris, CABG, PCI, aortic aneurysm, baseline atrial fibrillation/flutter, stroke, intermittent claudication, pacemaker, ICD, apolipoprotein A-1 and -B, creatinine, alanine aminotransferase, creatinine kinase, triglyceride level, CRP, antiarrhythmic treatment. log (NT-proBNP) |
| **Qureshi AI et al. 2006** | √ | √ | √ | √ | √ | √ | √ |  | Race/ethnicity |
| **Razvi S et al. 2010** | √ | √ | √ | √ | √ | √ | √ | √ | Social class, history of cerebrovascular disease, thyroid antibody status |
| **Rhee CM (dialysis). 2013** | √ | √ |  |  |  |  | √ |  | Race, hospitalization for noncardiovascular indication in the preceding year |
| **Rhee CM (NHANES III). 2013** | √ | √ | √ | √ | √ | √ | √ |  | Race, previous stroke, previous MI, pre-existing chronic HF (omitted from models stratified on chronic HF), eGFR, amiodarone, ACEI, β-blockers, calcium channel blockers (dihydropyridine and nondihydropyridine), digoxin, thiazide and loop diuretics, antihyperlipidemics (statins, niacin, and gemfibrozil), and long-acting nitrates |
| **Rhee CM et al. 2015** | √ | √ | √ |  | √ | √ | √ |  | Race/ethnicity, cause of end-stage renal disease, vascular access, dialysis vintage, congestive heart failure, cerebrovascular disease, MI, other cardiac disease, peripheral vascular disease, entry quarter, serum creatinine, albumin, ferritin, iron saturation, total iron binding capacity, bicarbonate, hemoglobin, calcium, phosphate, parathyroid hormone, spkt/V, weekly erythropoietin stimulating agent dose, and cumulative quarterly IV iron dose |
| **Rodondi N et al.2005** | √ | √ |  | √ | √ | √ | √ | √ | Race, prevalent CVD, poor or fair health, creatinine level, education, income, use of ACEI |
| **Sathyapalan^b^et al .2010** | √ | √ |  |  |  |  |  |  | Other covariates |
| **Schultz^c^ et al. 2011** | √ | √ |  | √ |  | √ | √ |  |  |
| **Selmer C et al. 2012** | √ | √ |  |  |  |  |  |  | Calendar year, Charlson comorbidity index, and socioeconomic status. |
| **Selmer C et al. 2014** | √ | √ |  |  |  |  |  |  | Calendar year |
| **Sgarbi JA et al. 2010** | √ | √ | √ | √ | √ | √ | √ |  | Cardiovascular disease |
| **Sharma AK et al. 2015** |  | √ |  |  |  |  | √ |  | Creatinine, digoxin, ACEI use |
| **Thvilum M (type). 2013** |  |  |  |  |  |  |  |  | The Charlson score |
| **Thvilum. (excess)**  **2013** |  |  |  |  |  |  |  |  | The Charlson score |
| **Tseng FY et al. 2012** | √ | √ | √ | √ | √ | √ | √ |  | Alcohol consumption, betel nut chewing, physical activity status, income, education level |
| **van de Ven AC et al. 2014** | √ | √ | √ | √ | √ | √ | √ |  | CVD, cancer, DVT, asthma/ COPD, RA, renal and/or liver disease. |
| **Walsh JP et al. 2005** | √ | √ | √ | √ | √ | √ | √ |  | CHD at baseline, hypertension treatment, exercise, and self-reported thyroid disease or goiter at baseline. |
| **Wang W et al. 2015** | √ | √ |  | √ | √ |  | √ |  | Anemia, renal dysfunction |
| **Waring (MrOS). 2012** | √ | All males |  | √ |  |  |  | √ | Race, clinic site, history of non-skin cancer, and self-reported health status |
| **Waring AC (CHS All Stars) 2012** | √ | √ |  |  |  |  |  |  | Race |
| **Yeap BB et al. 2013** | √ | All males | √ | √ | √ | √ |  |  | Creatinine level, medical comorbidity |
| **Zhang M et al. 2016** | √ | √ |  | √ | √ | √ | √ |  | Family of CAD, renal failure, heart failure, history of MI, number of diseased vessels, stent type, aspirin, β-lockers, ACEI and statins. |

^a^In the study by Gussekloo et al, the risk estimate of cardiovascular mortality only adjusted for sex.

^b^In the study by Sathyapalan et al, the risk estimate of cardiovascular mortality did not adjust for any risk factors.

^c^In the study by Schultz et al, the risk estimate of stroke adjusted for sex, age, and AF.

ACEI, angiotensin-converting enzyme inhibitors; AF, atrial fibrillation; ARB, angiotensin receptor antagonist; BMI, body mass index; CABG, coronary artery bypass grafting; CAD, coronary artery disease; CHD, coronary heart disease; COPD, chronic obstructive pulmonary disease; CRF, chronic renal failure; CRP, c-reactive protein; CVA, cerebral vascular asthma; CVD, cardiovascular disease; DVT, deep vein thrombosis; FT3, free triiodothyronine; FT4, free thyroxine; GFR, glomerular filtration rate; HF, heart failure; HIV, human immunodeficiency virus; ICD, implantable cardioverter defibrillator; IHD, ischemia heart disease; IL-6, interleukin-6; LVEF, left ventricular ejection fraction; MI, myocardial infarction; NYHA, New York Heart Association; PCI, percutaneous coronary intervention; RA, rheumatoid arthritis; TNF, tumor necrosis factor; TSH, thyroid-stimulating hormone.

| **Table S4: Additional subgroup analyses of main outcomes by TSH levels in SCH** | | | | | | | | | |
| --- | --- | --- | --- | --- | --- | --- | --- | --- | --- |
| **Factors** | **IHD** | | | |  | **Cardiac mortality** | | | |
|  | **N**  **studies** | **RR**  **(95%CI)** | **I^2^** | **P**  **value^a^** |  | **N**  **Studies** | **RR**  **(95%CI)** | **I^2^** | **P**  **Value^a^** |
| **SCH**  **TSH<10.0**  **TSH≥10.0** | 5  5 | 1.03(0.87-1.23)  1.32(1.00-1.74) | 42.9%  22.3% | 0.14  0.27 |  | -  - | -  - | -  - | -  - |
|  | **Cardiovascular mortality** | | | | | **All-cause mortality** | | | |
| **SCH**  **TSH<10.0**  **TSH≥10.0** | 5  5 | 1.15(0.91-1.45)  1.23(0.67-2.27) | 5.5%  24.1% | 0.38  0.26 | | 5  5 | 1.10(0.89-1.37)  1.07(0.72-1.58) | 69.1%  38.0% | 0.01  0.17 |

^a^P value for heterogeneity.

CI=confidence interval; IHD=ischemia heart disease; RR=relative risk; SCH=subclinical hypothyroidism; TSH=thyroid stimulating hormone.

| **Table S5. Subgroup analysis of risk of heart failure by mean age** | | | | | |
| --- | --- | --- | --- | --- | --- |
| **Mean age** | **N**  **Studies** | **Events/**  **participants** | **RR**  **(95%CI)** | **I^2^** | **P**  **Value^a^** |
| **≥80 yrs**  **65-79 yrs**  **<65 yrs** | -  6  2 | -  3102/20113  19931/579763 | -  1.21(0.97-1.50)  1.03(0.95-1.11) | -  61.4%  0.0% | -  0.37  Reference |

^a^P values indicate whether the pooled estimate in each subgroup differs from a nominated reference subgroup.

CI=confidence interval; RR=relative risk;

**Figure S1. Funnel plots for publication bias of included cohort studies.**

**
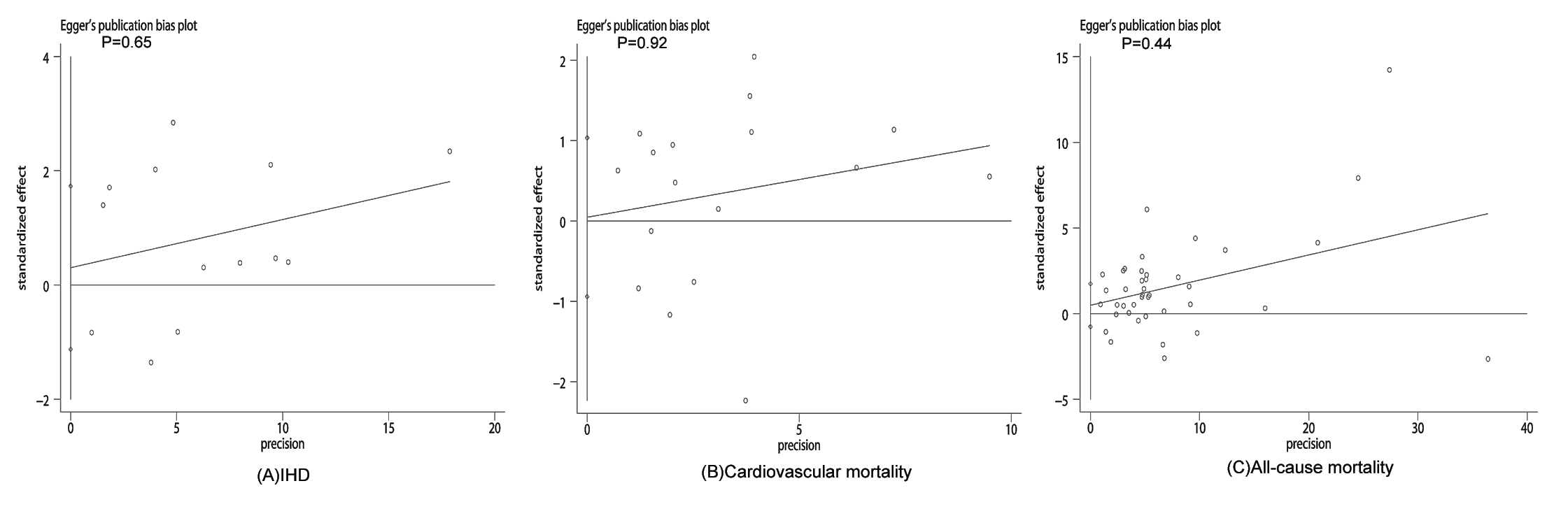
**

IHD=ischemia heart disease.

**Supplementary References**

1. Aho K, Gordin A, Palosuo T, Punsar S, Valkeila E, Karvonen M, et al. Thyroid autoimmunity and cardiovascular diseases. Eur Heart J. 1984;5(1):43-46.

2. Asvold BO, Bjoro T, Platou C, Vatten LJ. Thyroid function and the risk of coronary heart disease: 12-year follow-up of the HUNT study in Norway. Clin Endocrinol (Oxf). 2012;77(6):911-917.

3. Bai MF, Gao CY, Yang CK, Wang XP, Liu J, Qi DT, et al. Effects of thyroid dysfunction on the severity of coronary artery lesions and its prognosis. J Cardiol. 2014;64(6):496-500.

4. Bauer DC, Rodondi N, Stone KL, Hillier TA. Thyroid hormone use, hyperthyroidism and mortality in older women. Am J Med. 2007;120(4):343-349.

5. Boekholdt SM, Titan SM, Wiersinga WM, Chatterjee K, Basart DC, Luben R, et al. Initial thyroid status and cardiovascular risk factors: the EPIC-Norfolk prospective population study. Clin Endocrinol (Oxf). 2010;72(3):404-410.

6. Bruere H, Fauchier L, Bernard Brunet A, Pierre B, Simeon E, Babuty D, et al. History of thyroid disorders in relation to clinical outcomes in atrial fibrillation. Am J Med. 2015;128(1):30-37.

7. Ceresini G, Ceda GP, Lauretani F, Maggio M, Usberti E, Marina M, et al. Thyroid status and 6-year mortality in elderly people living in a mildly iodine-deficient area: the aging in the Chianti Area Study. J Am Geriatr Soc. 2013;61(6):868-874.

8. Chen HS, Wu TE, Jap TS, Lu RA, Wang ML, Chen RL, et al. Subclinical hypothyroidism is a risk factor for nephropathy and cardiovascular diseases in Type 2 diabetic patients. Diabet Med. 2007;24(12):1336-1344.

9. Chen S, Shauer A, Zwas DR, Lotan C, Keren A, Gotsman I. The effect of thyroid function on clinical outcome in patients with heart failure. Eur J Heart Fail. 2014;16(2):217-226.

10. de Jongh RT, Lips P, van Schoor NM, Rijs KJ, Deeg DJ, Comijs HC, et al. Endogenous subclinical thyroid disorders, physical and cognitive function, depression, and mortality in older individuals. Eur J Endocrinol. 2011;165(4):545-554.

11. Drechsler C, Schneider A, Gutjahr-Lengsfeld L, Kroiss M, Carrero JJ, Krane V, et al. Thyroid function, cardiovascular events, and mortality in diabetic hemodialysis patients. Am J Kidney Dis. 2014;63(6):988-996.

12. Frey A, Kroiss M, Berliner D, Seifert M, Allolio B, Guder G, et al. Prognostic impact of subclinical thyroid dysfunction in heart failure. Int J Cardiol. 2013;168(1):300-305.

13. Giri A, Edwards TL, LeGrys VA, Lorenz CE, Funk MJ, Schectman R, et al. Subclinical hypothyroidism and risk for incident ischemic stroke among postmenopausal women. Thyroid. 2014;24(8):1210-1217.

14. Grossman A, Weiss A, Koren-Morag N, Shimon I, Beloosesky Y, Meyerovitch J. Subclinical Thyroid Disease and Mortality in the Elderly: A Retrospective Cohort Study. Am J Med. 2016;129(4):423-430.

15. Gussekloo J, van Exel E, de Craen AJ, Meinders AE, Frolich M, Westendorp RG. Thyroid status, disability and cognitive function, and survival in old age. Jama. 2004;292(21):2591-2599.

16. Hak AE, Pols HA, Visser TJ, Drexhage HA, Hofman A, Witteman JC. Subclinical hypothyroidism is an independent risk factor for atherosclerosis and myocardial infarction in elderly women: the Rotterdam Study. Ann Intern Med. 2000;132(4):270-278.

17. Hsu JC, Li Y, Marcus GM, Hsue PY, Scherzer R, Grunfeld C, et al. Atrial fibrillation and atrial flutter in human immunodeficiency virus-infected persons: incidence, risk factors, and association with markers of HIV disease severity. J Am Coll Cardiol. 2013;61(22):2288-2295.

18. Hyland KA, Arnold AM, Lee JS, Cappola AR. Persistent subclinical hypothyroidism and cardiovascular risk in the elderly: the cardiovascular health study. J Clin Endocrinol Metab. 2013;98(2):533-540.

19. Iervasi G, Molinaro S, Landi P, Taddei MC, Galli E, Mariani F, et al. Association between increased mortality and mild thyroid dysfunction in cardiac patients. Arch Intern Med. 2007;167(14):1526-1532.

20. Imaizumi M, Akahoshi M, Ichimaru S, Nakashima E, Hida A, Soda M, et al. Risk for ischemic heart disease and all-cause mortality in subclinical hypothyroidism. J Clin Endocrinol Metab. 2004;89(7):3365-3370.

21. Karch A, Thomas SL. Autoimmune thyroiditis as a risk factor for stroke: a historical cohort study. Neurology. 2014;82(18):1643-1652.

22. Kim EJ, Lyass A, Wang N, Massaro JM, Fox CS, Benjamin EJ, et al. Relation of hypothyroidism and incident atrial fibrillation (from the Framingham Heart Study). Am Heart J. 2014;167(1):123-126.

23. Kim TH, Choi HS, Bae JC, Moon JH, Kim HK, Choi SH, et al. Subclinical hypothyroidism in addition to common risk scores for prediction of cardiovascular disease: a 10-year community-based cohort study. Eur J Endocrinol. 2014;171(5):649-657.

24. Laulund AS, Nybo M, Brix TH, Abrahamsen B, Jorgensen HL, Hegedus L. Duration of thyroid dysfunction correlates with all-cause mortality. the OPENTHYRO Register Cohort. PLoS One. 2014;9(10):e110437.

25. LeGrys VA, Funk MJ, Lorenz CE, Giri A, Jackson RD, Manson JE, et al. Subclinical hypothyroidism and risk for incident myocardial infarction among postmenopausal women. J Clin Endocrinol Metab. 2013;98(6):2308-2317.

26. Li X, Yang X, Wang Y, Ding L, Wang J, Hua W. The prevalence and prognostic effects of subclinical thyroid dysfunction in dilated cardiomyopathy patients: a single-center cohort study. J Card Fail. 2014;20(7):506-512.

27. Lin YC, Lin YC, Chen TW, Yang WC, Lin CC. Abnormal thyroid function predicts mortality in patients receiving long-term peritoneal dialysis: a case-controlled longitudinal study. J Chin Med Assoc. 2012;75(2):54-59.

28. McQuade C, Skugor M, Brennan DM, Hoar B, Stevenson C, Hoogwerf BJ. Hypothyroidism and moderate subclinical hypothyroidism are associated with increased all-cause mortality independent of coronary heart disease risk factors: a PreCIS database study. Thyroid. 2011;21(8):837-843.

29. Mitchell JE, Hellkamp AS, Mark DB, Anderson J, Johnson GW, Poole JE, et al. Thyroid function in heart failure and impact on mortality. JACC Heart Fail. 2013;1(1):48-55.

30. Molinaro S, Iervasi G, Lorenzoni V, Coceani M, Landi P, Srebot V, et al. Persistence of mortality risk in patients with acute cardiac diseases and mild thyroid dysfunction. Am J Med Sci. 2012;343(1):65-70.

31. Nanchen D, Gussekloo J, Westendorp RG, Stott DJ, Jukema JW, Trompet S, et al. Subclinical thyroid dysfunction and the risk of heart failure in older persons at high cardiovascular risk. J Clin Endocrinol Metab. 2012;97(3):852-861.

32. Parle JV, Maisonneuve P, Sheppard MC, Boyle P, Franklyn JA. Prediction of all-cause and cardiovascular mortality in elderly people from one low serum thyrotropin result: a 10-year cohort study. Lancet. 2001;358(9285):861-865.

33. Perez AC, Jhund PS, Stott DJ, Gullestad L, Cleland JG, van Veldhuisen DJ, et al. Thyroid-stimulating hormone and clinical outcomes: the CORONA trial (controlled rosuvastatin multinational study in heart failure). JACC Heart Fail. 2014;2(1):35-40.

34. Qureshi AI, Suri FK, Nasar A, Kirmani JF, Divani AA, Giles WH. Free thyroxine index and risk of stroke: results from the National Health and Nutrition Examination Survey Follow-up Study. Med Sci Monit. 2006;12(12):Cr501-506.

35. Razvi S, Weaver JU, Vanderpump MP, Pearce SH. The incidence of ischemic heart disease and mortality in people with subclinical hypothyroidism: reanalysis of the Whickham Survey cohort. J Clin Endocrinol Metab. 2010;95(4):1734-1740.

36. Rhee CM, Alexander EK, Bhan I, Brunelli SM. Hypothyroidism and mortality among dialysis patients. Clin J Am Soc Nephrol. 2013;8(4):593-601.

37. Rhee CMNI, Curhan GC, Alexander EK, Bhan I, Brunelli SM. Subclinical hypothyroidism and survival: the effects of heart failure and race. J Clin Endocrinol Metab. 2013;98(6):2326-2336.

38. Rhee CM, Kim S, Gillen DL, Oztan T, Wang J, Mehrotra R, et al. Association of thyroid functional disease with mortality in a national cohort of incident hemodialysis patients. J Clin Endocrinol Metab. 2015;100(4):1386-1395.

39. Rodondi N, Newman AB, Vittinghoff E, de Rekeneire N, Satterfield S, Harris TB, et al. Subclinical hypothyroidism and the risk of heart failure, other cardiovascular events, and death. Arch Intern Med. 2005;165(21):2460-2466.

40. Sathyapalan T, Manuchehri AM, Rigby AS, Atkin SL. Subclinical hypothyroidism is associated with reduced all-cause mortality in patients with type 2 diabetes. Diabetes Care. 2010;33(3):e37.

41. Schultz M, Kistorp C, Raymond I, Dimsits J, Tuxen C, Hildebrandt P, et al. Cardiovascular events in thyroid disease: a population based, prospective study. Horm Metab Res. 2011;43(9):653-659.

42. Selmer C, Olesen JB, Hansen ML, Lindhardsen J, Olsen AM, Madsen JC, et al. The spectrum of thyroid disease and risk of new onset atrial fibrillation: a large population cohort study. Bmj. 2012;345:e7895.

43. Selmer C, Olesen JB, Hansen ML, von Kappelgaard LM, Madsen JC, Hansen PR, et al. Subclinical and overt thyroid dysfunction and risk of all-cause mortality and cardiovascular events: a large population study. J Clin Endocrinol Metab. 2014;99(7):2372-2382.

44. Sgarbi JA, Matsumura LK, Kasamatsu TS, Ferreira SR, Maciel RM. Subclinical thyroid dysfunctions are independent risk factors for mortality in a 7.5-year follow-up: the Japanese-Brazilian thyroid study. Eur J Endocrinol. 2010;162(3):569-577.

45. Sharma AK, Vegh E, Orencole M, Miller A, Blendea D, Moore S, et al. Association of hypothyroidism with adverse events in patients with heart failure receiving cardiac resynchronization therapy. Am J Cardiol. 2015;115(9):1249-1253.

46. Thvilum M, Brandt F, Almind D, Christensen K, Brix TH, Hegedus L. Type and extent of somatic morbidity before and after the diagnosis of hypothyroidism. a nationwide register study. PLoS One. 2013;8(9):e75789.

47. Thvilum M, Brandt F, Almind D, Christensen K, Hegedus L, Brix TH. Excess mortality in patients diagnosed with hypothyroidism: a nationwide cohort study of singletons and twins. J Clin Endocrinol Metab. 2013;98(3):1069-1075.

48. Tseng FY, Lin WY, Lin CC, Lee LT, Li TC, Sung PK, et al. Subclinical hypothyroidism is associated with increased risk for all-cause and cardiovascular mortality in adults. J Am Coll Cardiol. 2012;60(8):730-737.

49. van de Ven AC, Netea-Maier RT, de Vegt F, Ross HA, Sweep FC, Kiemeney LA, et al. Associations between thyroid function and mortality: the influence of age. Eur J Endocrinol. 2014;171(2):183-191.

50. Walsh JP, Bremner AP, Bulsara MK, O'Leary P, Leedman PJ, Feddema P, et al. Subclinical thyroid dysfunction as a risk factor for cardiovascular disease. Arch Intern Med. 2005;165(21):2467-2472.

51. Wang W, Guan H, Gerdes AM, Iervasi G, Yang Y, Tang YD. Thyroid Status, Cardiac Function, and Mortality in Patients With Idiopathic Dilated Cardiomyopathy. J Clin Endocrinol Metab. 2015;100(8):3210-3218.

52. Waring AC, Harrison S, Samuels MH, Ensrud KE, Le BES, Hoffman AR, et al. Thyroid function and mortality in older men: a prospective study. J Clin Endocrinol Metab. 2012;97(3):862-870.

53. Waring AC, Arnold AM, Newman AB, Buzkova P, Hirsch C, Cappola AR. Longitudinal changes in thyroid function in the oldest old and survival: the cardiovascular health study all-stars study. J Clin Endocrinol Metab. 2012;97(11):3944-3950.

54. Yeap BB, Alfonso H, Hankey GJ, Flicker L, Golledge J, Norman PE, et al. Higher free thyroxine levels are associated with all-cause mortality in euthyroid older men: the Health In Men Study. Eur J Endocrinol. 2013;169(4):401-408.

55. Zhang M, Sara JD, Matsuzawa Y, Gharib H, Bell MR, Gulati R, et al. Clinical outcomes of patients with hypothyroidism undergoing percutaneous coronary intervention. Eur Heart J. 2016;37(26):2055-2065.
